# Supplementary material for: Adaptive deep brain stimulation as advanced Parkinson’s disease treatment (ADAPT study): protocol for a pseudo-randomised clinical study
Source: BMJ Open. 2019 Jun 14;9(6):e029652. doi: 10.1136/bmjopen-2019-029652 (PMC6575861; doi:10.1136/bmjopen-2019-029652)
Supplement: Author's manuscript [file bmjopen-2019-029652.draft_revisions.pdf]

BMJ Open is committed to open peer review. As part of this commitment we make the peer review history of every article we publish publicly available.

When an article is published we post the peer reviewers' comments and the authors' responses online. We also post the versions of the paper that were used during peer review. These are the versions that the peer review comments apply to.

The versions of the paper that follow are the versions that were submitted during the peer review process. They are not the versions of record or the final published versions. They should not be cited or distributed as the published version of this manuscript.

BMJ Open is an open access journal and the full, final, typeset and author-corrected version of record of the manuscript is available on our site with no access controls, subscription charges or pay-per-view fees (http://bmjopen.bmj.com).

If you have any questions on BMJ Open's open peer review process please email info.bmjopen@bmj.com

BMJ Open

Adaptive Deep Brain Stimulation as Advanced Parkinson’s disease Treatment (ADAPT study): protocol for a pseudo-randomised clinical study.

Journal:	*BMJ Open*
Manuscript ID	bmjopen-2019-029652
Article Type:	Protocol
Date Submitted by the Author:	04-Feb-2019

Complete List of Authors:	Piña-Fuentes, Dan; University Medical Centre Groningen, Neurosurgery; University Medical Center Groningen<br>Beudel, Martijn; University Medical Center Groningen, Neurology<br>Little, Simon; Institute of Neurology, Queen Square, London, Sobell Department of Motor Neuroscience and Movement Disorders<br>Brown, Peter; University of Oxford, Medical Research Council Brain Network Dynamics Unit<br>Oterdoom, D.L.Marinus; University Medical Center Groningen, Neurosurgery<br>van Dijk, J. Marc C.; University Medical Center Groningen, Neurosurgery

Keywords:	Parkinson-s disease < NEUROLOGY, Adaptive Deep Brain Stimulation, beta oscillations, subthalamic nucleus, internal globus pallidus, closed-loop

SCHOLARONE™
Manuscripts

Adaptive Deep Brain Stimulation as Advanced Parkinson's disease Treatment (ADAPT study): protocol for a pseudo-randomised clinical study.

D.A.I. Piña Fuentes MD[1]

M. Beudel MD PhD[2]

Simon Little MBBS, DPhil[3]

P. Brown MD PhD[4]

D. L. M. Oterdoom MD[1]

J.M.C. van Dijk MD PhD[1]

1. Department of Neurosurgery, University Medical Center Groningen, University of Groningen, Groningen, Netherlands

2. Department of Neurology, University Medical Center Groningen, University of Groningen, Groningen, Netherlands

3. Sobell Department of Motor Neuroscience and Movement Disorders, Institute of Neurology, Queen Square, London, UK

4. Medical Research Council Brain Network Dynamics Unit at the University of Oxford, Oxford, UK

Running title: ADAPT study protocol

Corresponding author:

D. Piña-Fuentes MD

Department of Neurosurgery,

University Medical Center Groningen,

Hanzeplein 1,

P. O. Box 30.001,

9700 RB Groningen,

The Netherlands.

tel: +31 50 361 0522

E-mail: d.a.i.pina.fuentes@umcg.nl

Word count

Abstract: 298

Full text: 3700

Number of figures: 4

Number of references: 29

Keywords:

Parkinson's disease, adaptive deep brain stimulation, subthalamic nucleus, internal globus pallidus, local field potentials, beta oscillations, closed-loop.

ABSTRACT

Introduction

Adaptive deep brain stimulation (aDBS), based on the detection of increased beta oscillations in the subthalamic nucleus (STN), has been assessed in Parkinson's disease (PD) patients, during the immediate postoperative setting. In these studies, aDBS was shown to be at least as effective as conventional DBS (cDBS), whilst stimulation time and side effects were reduced. However, the effect of aDBS on motor symptoms and stimulation-induced side effects, during the chronically implanted phase (after the stun effect of DBS placement has disappeared) has not yet been determined.

Methods and analysis

This protocol describes a single-centre clinical study in which aDBS will be tested in 14 PD patients, with electrodes implanted either in the STN or the internal globus pallidus, undergoing battery replacement. Patients included will be allocated in a pseudo-randomised fashion to a 3-condition (no stimulation/ cDBS / aDBS), crossover design. A battery of tests will be conducted and recorded during each condition, which are aimed to measure the severity of motor symptoms and side effects. These tests include a tablet-based tapping test, a subscale of the Movement Disorder Society- unified Parkinson's disease rating scale (subMDS-UPDRS), the Speech Intelligibility Test (SIT) and a tablet-based version of the Stroop Test. SubMDS-UPDRS and SIT recordings will be blindly assessed by independent raters. Data will be analysed using a generalised linear mixed-effects model.

Ethics and dissemination: this protocol was approved by the Ethical Committee of the Institution in which the study will be carried out (University Medical Centre Groningen). Data management and compliance to research policies and standards of our centre, including data privacy, storage and veracity, will be controlled by an independent monitor. All the scientific findings derived from this protocol are aimed to be made public through publication of articles in international journals.

Trial registration number: NTR 5456

Strengths and limitations of this study

The results of this protocol will be able to strengthen the evidence regarding the effectiveness of aDBS systems.

Subjects to be included have had their DBS electrodes chronically, and therefore the stun effect is not present in this patient population.

Intraoperative testing will minimise the time that electrodes are externalized and that patients are exposed to potential risks of infection.

Testing and washout periods will be limited by the intraoperative time available for each patient.

The intraoperative setting will limit the tests available to be assessed in PD patients.

INTRODUCTION

Background and rationale.

Deep brain stimulation (DBS) is currently one of the most effective advanced treatments for patients with Parkinson's disease (PD). At present, conventional DBS (cDBS) provides the continuous delivery of fixed-frequency and -amplitude electrical pulses through the electrodes, implanted mostly in either the subthalamic nucleus (STN) or the internal globus pallidus (GPi).[1,2] Although cDBS is an effective treatment, it still has limitations in terms of efficacy, side effects and efficiency, and these

have not undergone substantial improvements since the implementation of cDBS in clinical practice, approximately 20 years ago.[3] However, new DBS-systems directed to improve current stimulation algorithms are currently under development. These systems are often referred to as adaptive DBS (aDBS), as they aim to modulate the delivery of electrical stimulation, controlled by the detection of symptoms, like tremor, or aberrant signals (biomarkers) that have been implicated in the pathophysiology of the disease.[4] The ultimate goal of aDBS-systems is to reduce the total amount of stimulation delivered, in a way in which beneficial effects of DBS are enhanced, while side effects are reduced.[5] Furthermore, decreasing the total amount of stimulation delivered will allow to extend the battery life(span) of DBS devices.

Several biomarkers in PD have been proposed for use as a feedback signal for aDBS.[6] Currently, the presence of prominent beta oscillations (13-35 Hz), in either the STN or the GPi, seems to be the most promising, since it correlates with the severity of bradykinesia and rigidity.[7] aDBS-systems based on beta oscillations have thus far been tested in the immediate postoperative setting. Evidence to date suggests that aDBS may be at least as effective as cDBS systems, whilst stimulation time and side effects are reduced.[8–10] Although aDBS has been evaluated mostly with regard to the STN, preliminary evidence indicates that aDBS could be equally effective when applied to the GPi.[11] The correlation of other prominent PD symptoms (such as tremor) with beta oscillations is far less robust. Therefore, the impact of aDBS on these symptoms is yet to be addressed.[12,13]

Besides its effect on motor symptoms, a common side effect that could potentially be addressed by aDBS is stimulation-induced dysarthria. Preliminary data in the immediate postoperative phase showed that aDBS did not induce dysarthria, in contrast to cDBS.[14] Likewise, due to the reduction in the total amount of stimulation that occurs in aDBS, this new technology could potentially help to tackle non-motor side effects induced by stimulation. Certain psychiatric symptoms caused by dopaminergic therapy, such as impulsive behaviour, can be improved by the medication reduction that cDBS allows. On the other hand, stimulation can paradoxically induce impulsive behaviour.[15,16]. It was first proposed that this side effect was due to unintended stimulation of limbic areas of the STN.[17] However, it has been shown recently that DBS has a positive effect on risk-reward decision behaviour in PD patients, and that such an effect was mostly dependent on stimulation of the motor part of the STN.[18] This finding indicates that at least some of the non-motor effects of DBS are mediated through direct stimulation of the motor part of the STN. This is supported by increasing evidence that indicates that the motor and non-motor functions of the STN are not perfectly segregated.[19] Therefore, aDBS might be used as an additional option to address stimulation-induced side effects, such as impulsive behaviour, as it could provide a functional selectivity, by only triggering stimulation based on (primarily) motor biomarkers (e.g. beta oscillations).

Objectives.

The aim of this study is to assess whether aDBS provides a similar or better motor improvement compared to cDBS, while limiting the presence of stimulation-induced side effects in patients with chronically implanted electrodes. Stimulation efficacy is assessed using a battery of tests, aimed to measure the motor response to different conditions, and the severity of dysarthria and/or impulsivity. The goals of this protocol are summarized in three questions:

I. Is aDBS at least as efficacious as cDBS in terms of motor improvement?

II. Is the speech-related and cognitive side-effect profile of aDBS lower than that of cDBS?

III. What is the amount of stimulation provided in aDBS compared to cDBS?

Trial design.

This protocol describes an exploratory study in which PD-patients are allocated to a 3-condition (no stimulation (NoStim) / cDBS / aDBS), crossover design (Figure 1). The conditions are pseudo-randomised in a 3:2:2 format, in such a way that for every 3 patients who start with no-stimulation, 2 patients will have aDBS first, and 2 will have cDBS first. The main reason for this proposed pseudo-randomisation is to minimise the impact that an incomplete washout-effect derived from stimulation could have on the scores of NoStim condition, as this condition is the baseline for the comparison of the effect of the two stimulation modalities. In four patients, both stimulation conditions will be separated by a NoStim condition, to minimize the carryover effect that cDBS could have on aDBS (or vice versa). Lastly, NoStim will be the last condition in four patients, in order to partially balance the effects of training or fatigue on the tasks.

The performance of several tasks during the three conditions will place during the battery replacement surgery, when a temporal externalization of the DBS extension wires is performed. This means that subjects to be included have had their DBS electrodes implanted for at least 3-5 years, and therefore the stun effect is not present in this patient population. It was decided to perform this protocol intraoperatively, as it allows the surgery to be completed in one session, minimising the time that electrodes are externalized and patients are exposed to potential risks of infection.

METHODS AND ANALYSIS:

<u>Methods: Participants, interventions, and outcomes</u>

Study setting.

Single-centre study, at the University Medical Centre Groningen.

Inclusion criteria:

- PD patients with DBS electrodes implanted bilaterally in either the STN or GPi.
- Patients are cognitively capable of performing the tests included in this protocol. This item is subjectively evaluated during the patient interview, by looking at the performance of the candidate when completing a trial set of the tablet-based tests used during the experiment.
- Able to provide written informed consent.
- Physical condition that enables 20-30 minutes of testing. This is evaluated according to the clinical records, and the fragility of the patient (determined by the treating neurologist).
- Ability to undergo testing in the OFF dopaminergic state, determined by the clinical condition of the patient after medication withdrawal.

Exclusion criteria:

- Patients who cannot tolerate battery replacement under local anaesthesia (e.g. presence of severe OFF medication tremor, severe anxiety, etc.).
- <18 years old.
- Clinical disorders or diseases that could interfere with clinical assessment (e.g. vision impairment that cannot be corrected with glasses, severe limb movement restriction, etc.).
- Cognitive impairment sufficient to prevent the patient to consider and retain the necessary information to make a decision regarding the participation in the study.

For peer review only - http://bmjopen.bmj.com/site/about/guidelines.xhtml

Investigational device.

The responsive (closed loop) aDBS is delivered through a custom-made stimulation-amplifier which allows local field potentials (LFPs) to be recorded during the application of DBS. With this device, LFPs are filtered in real time around a subject specific beta frequency (±3 Hz around peak).[9] Afterwards, a median threshold is selected, in order to deliver monopolar stimulation every time that beta activity in the filtered LFP exceeds the predefined threshold. Stimulation pulses are charge-balanced and asymmetric, mirroring the waveform of the standard clinical Medtronic Activa PC. Pulses are delivered at 130 Hz with a pulse width 60 µs (standard clinical parameters), using a ramping period of 250 ms at onset and offset, in order to help limit the presence of paraesthesias, autonomic or other non-specific side effects, such as dizziness or nausea.[20] From the four contacts available for each electrode lead, 2 electrodes are required to perform bipolar LFP recordings (either contacts 0-2 or 1-3), while one electrode (in between the two recording electrodes) is left available for stimulation (either contacts 1 or 2). The stimulation-amplifier has been built to strict safety guidelines (Report 90 BS EN60601-1 safe design, construction of medical equipment) and extensively tested[8,9,11,21], and found to be safe and reliable.

Procedure.

Patients have to skip at least one medication dose, and are not allowed to be under the effect of prolonged-release dopaminergic medication, in order to be classified as OFF medication before surgery. Patients are brought to the operating theatre for their battery replacement procedure. After the battery is exposed, the old battery is detached from the DBS electrodes and explanted. At this moment, two temporary wires are attached to the DBS electrode extension cables and connected to a combined stimulator and amplifier (See previous section). A brief rest recording of 30-60 seconds is performed, from each of the two bipolar montages (0-2 or 1-3) available for each hemisphere. Power spectral density (PSD) estimates are obtained from each rest recording, and the bipolar contact pair (0-2 or 1-3) per hemisphere with the highest beta peak power is selected. Monopolar cDBS is then titrated, using the available contact located between the selected recording contacts. The voltage for monopolar stimulation is titrated initially based on an approximation of the voltage used in clinical practice for each individual patient (independently of the montage used in clinical practice). Voltage is increased or decreased according to the presence of side effects, clinical effect, and interference with LFP recordings (in practice, we have seen that voltages >3.5 V are more prone to cause interference with the recordings, and these are also rare stimulation parameters in clinical practice). Once an optimum stimulation voltage is established, the patients are assigned to each of 3 counter-balanced conditions in which the patient is either stimulated with aDBS or cDBS, or not stimulated. Stimulation parameters (voltage, frequency, pulse width and contact selection) remain constant for both aDBS and cDBS, differing only in the timing of stimulation delivered according to beta detection. As battery replacements are performed under local anaesthesia, we aim to limit the duration of the experiment to a maximum of 25-30 min.

Clinical tests and outcomes:

Each patient performs the following tasks in each of the three pseudo-randomised conditions:

a) Tablet-based tapping test: several analog and digital versions of the tapping test have been validated to objectively measure the presence of bradykinesia in PD patients.[22–24] We developed a custom tablet-based finger tapping test using the PsyPad platform.[25] In this test, a tablet is presented to the patient, in which two squares appear at the left and right side of the screen (Figure 2). The patient then has to press the squares in an alternating pattern. We tested this setup, as a proof of principle, on a PD patient who underwent battery replacement,[21] using 50 tapping

iterations per condition. In order to optimize the time available for the battery of tests, it was decided to reduce the iterations to 20. Since the duration of each iteration was approximately 500ms, 20 iterations (10 back-and-forth screen tapings) will be roughly equivalent to 10 finger tapings, which is the amount of taps required to measure tapping items from the Movement Disorder Society- unified Parkinson's disease rating scale (MDS-UPDRS).

b) Subscale of the MDS-UPDRS (subMDS-UPDRS)[26] (Part III, items 3.4, 3.5, 3.6, 3.15, 3.17 a, b): as patients are lying on the operating table during the experiment, only selected items can be performed by the patients. The items that are included and filmed are: finger tapping, hand movements, pronation-supination, rest tremor, and postural tremor.

c) Speech Intelligibility Test (SIT): this is a modified version of the Assessment of Intelligibility for the Dysarthric Speech.[27,28] In this test, the patient is instructed to read out a list of 18 sentences (approximately 110 words per condition), while the speech is recorded with a mobile app. The words that form each sentence are randomly chosen, taking into account that each sentence must follow the correct order of a proper grammatical construction. Voice recordings are sent to a speech therapist, who blindly evaluates the sentences and writes them down. Afterwards, the sentences written by the speech therapist are compared with the original list, and the amount of words that coincide are expressed as a percentage of the total words.

d) Tablet- based version of the Stroop Test:[29] in this test, 15 colour words are presented to each patient in a sequential order, and each word is coloured in (Figure 3). From those 15 words, 5 are coloured in the same colour as the word written (congruent), while 10 words are coloured in a colour different than the word written (incongruent). We chose an unbalanced design, as our main focus is the effect of stimulation on incongruent words between conditions, rather than the effect of stimulation between congruent and incongruent words. For each word presented, four buttons are presented at the bottom of the tablet screen, each containing different colour words. The patient has to select the button containing the colour word presented on the screen, independently of the colour in which the word is coloured in. Reaction times and accuracy for each trial are directly uploaded to an online database, from which they can be extracted for data analysis.

Order of the tests:

After the battery is exposed, and before the performance of the clinical tests can start, a number of preparations are required in order to program the aDBS (see *Procedure* section). This requirement can lead to individual differences in the time available to perform the clinical tests, due to the maximum amount of intraoperative time allowed for this experiment. Therefore, the sequence in which the clinical tests are performed on each condition is ordered according to the approximate time it takes to perform each of them, the order being as follows: 1. Tabled-based tapping test, 2. subMDS-UPDRS, 3. Speech and Intelligibility Test (SIT), 4. Table-based version of the Stroop Test. If during the course of each individual experiment it is considered that the time will not be sufficient for a patient to complete the full protocol, only the first test(s) could be selected to be performed, in order to ensure that at least one of the tests will be completed for each of the three conditions.

Sample size:

Based on pilot data (conducted in Oxford / London),[14] SIT scores turned out to be 15% better in aDBS than in cDBS with respective means of 60 and 71 (range 0 - 100) and with a common standard deviation of 14 (n= 6). Using an α of 0.05 and an β of 0.8 a power calculation shows a sample size of 11 to show a significant difference between aDBS and cDBS in our chronically implanted cohort. Due to the high chance of technical challenges given the experimental condition of recording in the

operating theatre under time pressure we have set the total number of inclusions to 14 patients with bilateral recordings to account for patient drop out.

Recruitment:

Patients who are scheduled for a battery replacement at the University Medical Centre Groningen are contacted by telephone 2 weeks prior to their hospital admission to be informed about the study. Complementary written information is sent by mail to patients who show an interest in participating in the study. At the time of hospital admission, each patient is personally approached by one of the members of the research team, if they meet all of the inclusion criteria and none of the exclusion criteria. Patients who decide to participate in the study will then be given the opportunity to ask further questions and invited to sign the consent form.

Methods: Assignment of interventions

Allocation:

The ultimate goal of the allocation procedure is to have a pseudo-randomisation in which all the possible combinations of the block design are covered. (i.e. 3:2:2, see *Trial Design* section, Figure 1). For that reason, patients will be sequentially allocated to each possible pseudo-randomisation scenario, until 14 patients are allocated. Only successful bilateral measurements will be considered in the pseudo-randomisation list. At first, a pseudo-randomisation of 3:1:1 (10 patients) will be set as the allocation goal, in order to ensure that all conditions of the pseudo-randomisation could be met by mid-2019. Depending on the number of aborted experiments and the deadline for inclusion, 4 patients more may potentially be included, to achieve the 3:2:2 pseudo-randomisation.

Blinding (masking)

Patients will be blinded to the stimulation condition. Video and voice recordings will be taken for the subMDS-UPDRS and SIT parts, and will be blindly evaluated by 2 neurologists experienced in movement disorders, and a speech therapist, respectively. Analysis of tablet based outcomes will be automated and matched across the three conditions.

Methods: Data collection, management, and analysis

Data management.

Data is stored on a central database using REDCap storage system, being accessible only to the authorized personnel involved in this study.

Statistical methods.

Simple scores from the subMDS-UPDRS, Tabled-based tapping test, SIT and Stroop Test during aDBS vs cDBS or NoStim will be checked for normality using Q-Q plots, and if normally distributed, will be compared using a generalised linear mixed-effects model. If the data is non-normally distributed a data transformation will be applied, or a distributions different than normal will be employed for the test.

Methods: Monitoring

Harms. The experimental procedure does not substantially expose patients to increased risks, as there are no extra surgical procedures required, and patients are stimulated only with less electrical current than in their conventional setting. Given that the experiments take place in the operation room, and only add less than 30 minutes to the total surgery duration, there is no significant added risk of infection. Since patients are tested in the OFF dopaminergic state, this exaggerates their symptoms to certain extent. However, DBS remains active until the moment of the surgery, which limits the impact of medication discontinuation. In previous aDBS studies, electrodes were exposed for hours or days,[9,10] without any increased risk of infection. Therefore, as battery replacement is completed in one session, the amount of time in which the extension wires are exposed is considerably shortened in this study. Nevertheless, infections will be considered an adverse event and infection rates monitored.

Monitoring. Data management and compliance to research policies and standards of our centre, including data privacy, storage and veracity, are verified by an independent monitor from a different department within the hospital. As this is an investigator-initiated study, the project leader submits, once a year throughout the clinical trial, a safety report to the accredited local ethics committee. Serious adverse events, defined by the Central Committee on Research Involving Human Subjects in the Netherlands as 'any untoward medical occurrence or effect that at any dose results in death, is life threatening (at the time of the event), requires hospitalization or prolongation of existing inpatients' hospitalization or results in persistent or significant disability or incapacity', will be promptly reported to the corresponding governmental authorities.

Patient and Public Involvement. In order to make sure that the experiments are as comfortable as possible for the subjects, a short Patient Reported Outcome (PRO) questionnaire will be conducted on the first five patients (Figure 4). This PRO consists of 5 questions in which patients can indicate whether the communication was adequate and empathetic and whether the procedure was not too demanding, uncomfortable or painful. The feedback of these reports will be used to evaluate the adequacy of the protocol, and changes will be incorporated into the following study procedures, if necessary via an amendment.

ETHICS AND DISSEMINATION:

Research ethics approval.

This protocol is approved by the local ethics committee of our institution, and all procedures are carried out according to the Declaration of Helsinki and the Dutch legislation on medical research involving human subjects.

Protocol amendments.

The protocol presented here is the 5th version (9th December, 2018) of the original protocol prepared for this study. The 2nd version of the protocol (15th September, 2015) got the approval of the ethical committee, after several modifications of the 1st version (22th July, 2015). In version 3 (12th April, 2017), an attempt was made to include patients undergoing new implantations. However, this was dropped in subsequent versions, since: a)patients undergoing new implantations were often considerably fatigued at the moment of the measurement and could not perform all the tasks; b) differences in total intraoperative time between new implantations and battery replacements meant that the time available for the measurement was considerably less during new implantations; c) the stereotactic frame slightly restricted the visual field of the patients in new implantations. Version 4

(7th September, 2017) and version 5 were meant to increase the total number of patients to be included, as several failed or incomplete measurements took place during the initial inclusions.

Consent

During the inclusion interview, patients are informed that the participation in this study is on an entirely voluntary basis, and that they are therefore allowed at any moment to withdraw their consent. No economic reward is provided for the participation in this study.

Confidentiality.

Only indirect personal data from each patient are stored as coded information, in order to prevent the possibility that patient data could be traced back to an individual patient. The coding list will be safeguarded in a secured facility located in our centre. Data derived from the clinical tests or recordings could be exported only as anonymized data to be used for statistical analysis.

Declaration of interests.

All authors declare to have no conflict of interests.

Access to data.

Only authorized personnel have access to the coding list and the software in which the data is stored.

Dissemination policy.

All the scientific findings derived from this protocol are aimed to be made public through publication of articles in international journals.

FUNDING STATEMENT: This study is publicly funded by grants received from the Dutch Brain Foundation ('Hersenstichting Nederland', grant F2015(1)-04), the National Mexican Council of Science and Technology (CONACYT) grant number CVU 652927, and the University of Groningen/University Medical Centre Groningen (RuG/UMCG). SL is personally supported by a Wellcome trust post-doctoral clinical research training grant (105804/Z/14/Z)

TRIAL REGISTRATION: NEDERLANDS TRIAL REGISTER – http://www.trialregister.nl

NTR NUMBER: 5456

AUTHOR CONTRIBUTIONS: MB and JMCvD designed and prepared the first draft of the protocol, and initiated the study. DPF formatted the protocol according to the SPIRIT guidelines and prepared the manuscript. SL, PB and DLMO critically reviewed the protocol procedures and manuscript. All authors reviewed and approved the final version of this manuscript.

REFERENCES:

1 Follett KA, Weaver FM, Stern M, *et al.* Pallidal versus subthalamic deep-brain stimulation for Parkinson's disease. *N Engl J Med* 2010;**362**:2077–91. doi:10.1056/NEJMoa0907083

2 Odekerken VJJ, Boel JA, Schmand BA, *et al.* GPi vs STN deep brain stimulation for Parkinson disease: Three-year follow-up. *Neurology* 2016;**86**:755–61. doi:10.1212/WNL.0000000000002401

3 Picillo M, Lozano AM, Kou N, *et al.* Programming Deep Brain Stimulation for Parkinson's Disease: The Toronto Western Hospital Algorithms. *Brain Stimul* 2016;**9**:425–37.

doi:10.1016/J.BRS.2016.02.004

4 Priori A, Foffani G, Rossi L, *et al.* Adaptive deep brain stimulation (aDBS) controlled by local field potential oscillations. *Exp Neurol* 2013;**245**:77–86. doi:10.1016/J.EXPNEUROL.2012.09.013

5 Beudel M, Brown P. Adaptive deep brain stimulation in Parkinson's disease. *Parkinsonism Relat Disord* 2016;**22 Suppl 1**:S123-6. doi:10.1016/j.parkreldis.2015.09.028

6 Hoang KB, Cassar IR, Grill WM, *et al.* Biomarkers and Stimulation Algorithms for Adaptive Brain Stimulation. *Front Neurosci* 2017;**11**:564. doi:10.3389/fnins.2017.00564

7 Neumann W-J, Degen K, Schneider G-H, *et al.* Subthalamic synchronized oscillatory activity correlates with motor impairment in patients with Parkinson's disease. *Mov Disord* 2016;**31**:1748–51. doi:10.1002/mds.26759

8 Little S, Pogosyan A, Neal S, *et al.* Adaptive Deep Brain Stimulation In Advanced Parkinson Disease. Ann. Neurol. 2013;**74**:449–57. doi:10.1002/ana.23951

9 Little S, Beudel M, Zrinzo L, *et al.* Bilateral adaptive deep brain stimulation is effective in Parkinson's disease. *J Neurol Neurosurg Psychiatry* 2016;**87**:717–21. doi:10.1136/jnnp-2015-310972

10 Arlotti M, Marceglia S, Foffani G, *et al.* Eight-hours adaptive deep brain stimulation in patients with Parkinson disease. *Neurology* 2018;**90**:e971–6. doi:10.1212/WNL.0000000000005121

11 Pina-Fuentes D, van Zijl JC, van Dijk JMC, *et al.* The characteristics of pallidal low-frequency and beta bursts could help implementing adaptive brain stimulation in the parkinsonian and dystonic internal globus pallidus. *Neurobiol Dis* 2019;**121**:47–57. doi:10.1016/j.nbd.2018.09.014

12 Qasim SE, de Hemptinne C, Swann NC, *et al.* Electrocorticography reveals beta desynchronization in the basal ganglia-cortical loop during rest tremor in Parkinson's disease. *Neurobiol Dis* 2016;**86**:177–86. doi:10.1016/j.nbd.2015.11.023

13 Beudel M, Oswal A, Jha A, *et al.* Oscillatory Beta Power Correlates With Akinesia-Rigidity in the Parkinsonian Subthalamic Nucleus. Mov. Disord. 2017;**32**:174–5. doi:10.1002/mds.26860

14 Little S, Tripoliti E, Beudel M, *et al.* Adaptive deep brain stimulation for Parkinson's disease demonstrates reduced speech side effects compared to conventional stimulation in the acute setting. J. Neurol. Neurosurg. Psychiatry. 2016;**87**:1388–9. doi:10.1136/jnnp-2016-313518

15 Kim A, Kim YE, Kim H-J, *et al.* A 7-year observation of the effect of subthalamic deep brain stimulation on impulse control disorder in patients with Parkinson's disease. *Parkinsonism Relat Disord* Published Online First: 20 July 2018. doi:10.1016/J.PARKRELDIS.2018.07.010

16 Abbes M, Lhommee E, Thobois S, *et al.* Subthalamic stimulation and neuropsychiatric symptoms in Parkinson's disease: results from a long-term follow-up cohort study. *J Neurol Neurosurg Psychiatry* 2018;**89**:836–43. doi:10.1136/jnnp-2017-316373

17 Winter C, Lemke C, Sohr R, *et al.* High frequency stimulation of the subthalamic nucleus modulates neurotransmission in limbic brain regions of the rat. *Exp brain Res* 2008;**185**:497–507. doi:10.1007/s00221-007-1171-1

18 Irmen F, Horn A, Meder D, *et al.* Sensorimotor subthalamic stimulation restores risk-reward trade-off in Parkinson's disease. *Mov Disord* 2018;:1–11. doi:10.1002/mds.27576

19 Eisenstein SA, Koller JM, Black KD, *et al.* Functional anatomy of subthalamic nucleus

stimulation in Parkinson disease. *Ann Neurol* 2014;**76**:279–95. doi:10.1002/ana.24204

20 Dayal V, Limousin P, Foltynie T. Subthalamic Nucleus Deep Brain Stimulation in Parkinson's Disease: The Effect of Varying Stimulation Parameters. *J Parkinsons Dis* 2017;**7**:235–45. doi:10.3233/JPD-171077

21 Piña-Fuentes D, Little S, Oterdoom M, *et al.* Adaptive DBS in a Parkinson's patient with chronically implanted DBS: A proof of principle. *Mov Disord* 2017;**32**. doi:10.1002/mds.26959

22 Bronte-stewart HM, Ding L, Alexander C, *et al.* Quantitative Digitography (QDG): A Sensitive Measure of Digital Motor Control in Idiopathic Parkinson ' s Disease. 2000;**15**:36–47.

23 Noyce AJ, Treacy C, Budu C, *et al.* The new Bradykinesia Akinesia Incoordination (BRAIN) test: preliminary data from an online test of upper limb movement. Mov. Disord. 2012;**27**:157–8. doi:10.1002/mds.23947

24 Kassavetis P, Saifee TA, Roussos G, *et al.* Developing a Tool for Remote Digital Assessment of Parkinson's Disease. *Mov Disord Clin Pract* 2016;**3**:59–64. doi:10.1002/mdc3.12239

25 Turpin A, Lawson DJ, McKendrick AM. PsyPad: a platform for visual psychophysics on the iPad. *J Vis* 2014;**14**:16. doi:10.1167/14.3.16

26 Goetz CG, Tilley BC, Shaftman SR, *et al.* Movement Disorder Society-sponsored revision of the Unified Parkinson's Disease Rating Scale (MDS-UPDRS): scale presentation and clinimetric testing results. *Mov Disord* 2008;**23**:2129–70. doi:10.1002/mds.22340

27 Yorkston KM, Beukelman DR. Communication efficiency of dysarthric speakers as measured by sentence intelligibility and speaking rate. *J Speech Hear Disord* 1981;**46**:296–301.

28 Tripoliti E, Zrinzo L, Martinez-Torres I, *et al.* Effects of contact location and voltage amplitude on speech and movement in bilateral subthalamic nucleus deep brain stimulation. *Mov Disord* 2008;**23**:2377–83. doi:10.1002/mds.22296

29 Stroop JR. Studies of interference in serial verbal reactions. *J Exp Psychol* 1935;**18**:643.

Figure 1. Trial design and pseudo-randomisation block.

Figure 2. Example of a trial of the tablet-based bradykinesia test.

Figure 3. Example of two trials of the tablet-based Stroop test with congruent (left) and incongruent (right) conditions.

Figure 4. Example of the Patient Reported Outcome questionnaire form conducted on the participants.

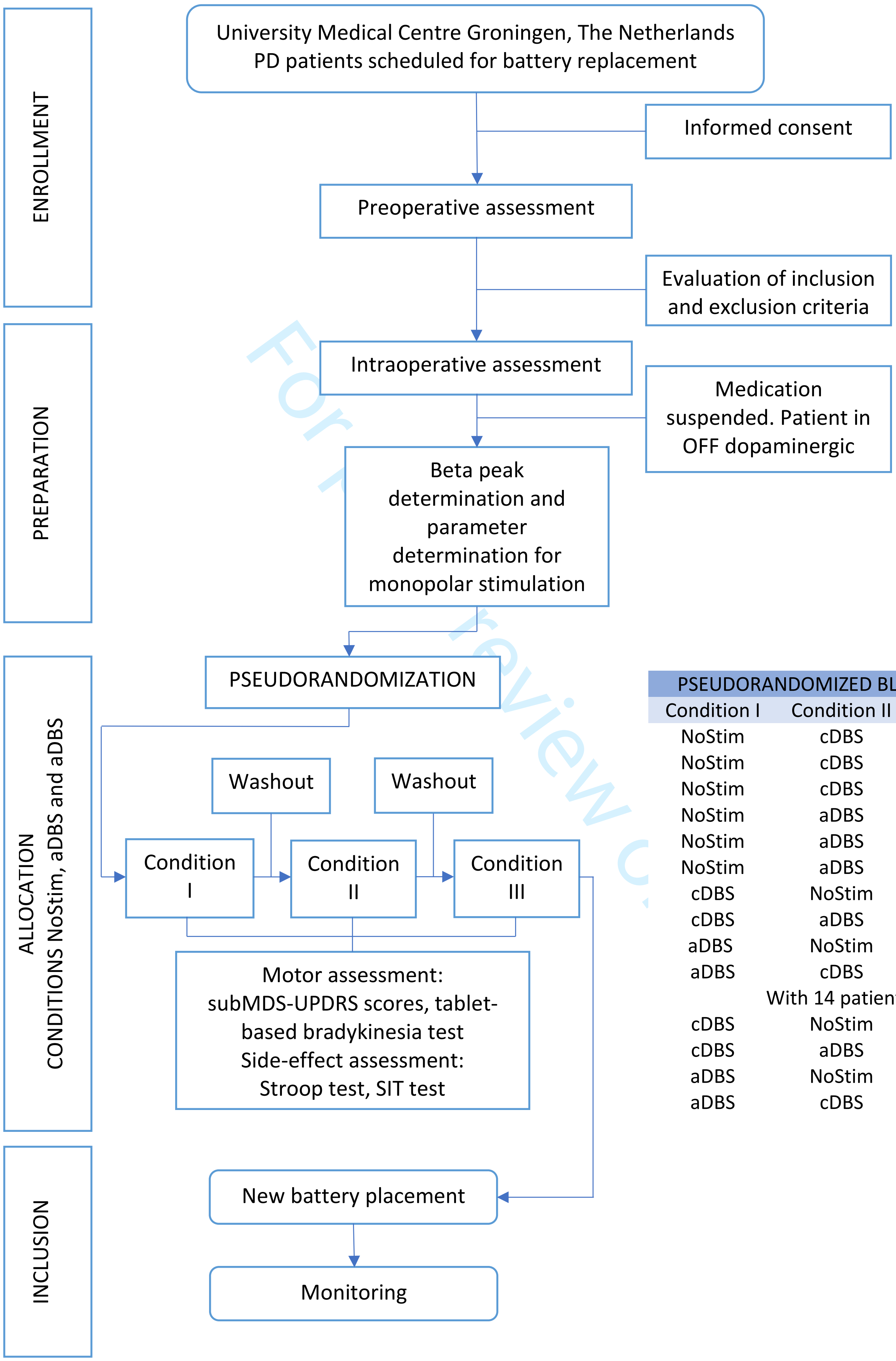

PSEUDORANDOMIZED BLOCK DESIGN		
Condition I	Condition II	Condition III
NoStim	cDBS	aDBS
NoStim	cDBS	aDBS
NoStim	cDBS	aDBS
NoStim	aDBS	cDBS
NoStim	aDBS	cDBS
NoStim	aDBS	cDBS
cDBS	NoStim	aDBS
cDBS	aDBS	NoStim
aDBS	NoStim	cDBS
aDBS	cDBS	NoStim
	With 14 patients:	
cDBS	NoStim	aDBS
cDBS	aDBS	NoStim
aDBS	NoStim	cDBS
aDBS	cDBS	NoStim

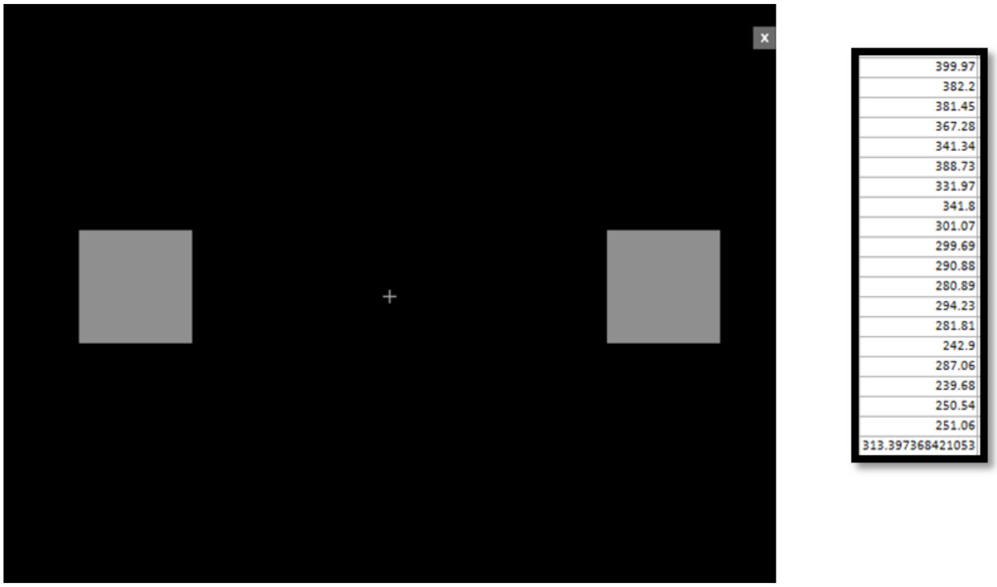

Figure 2. Example of a trial of the tablet-based bradykinesia test.

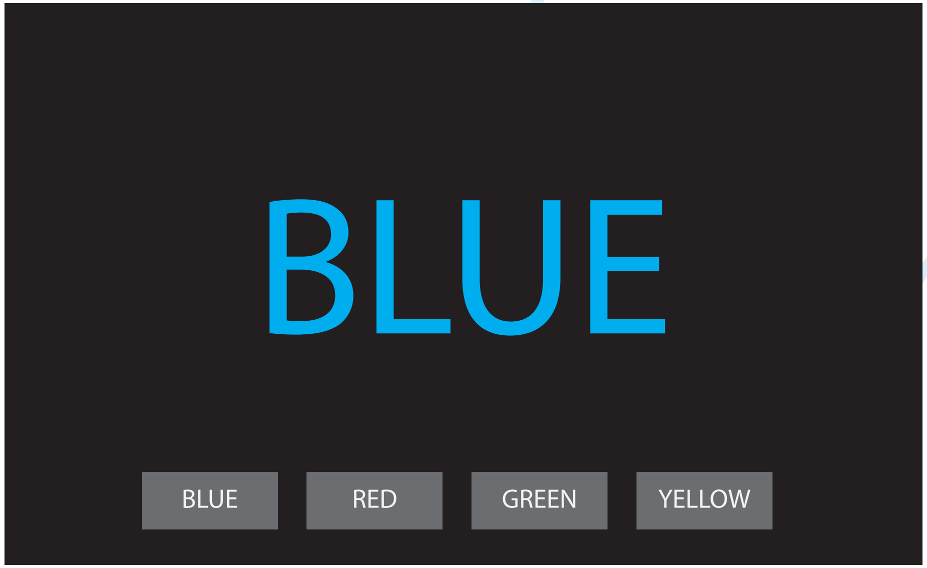

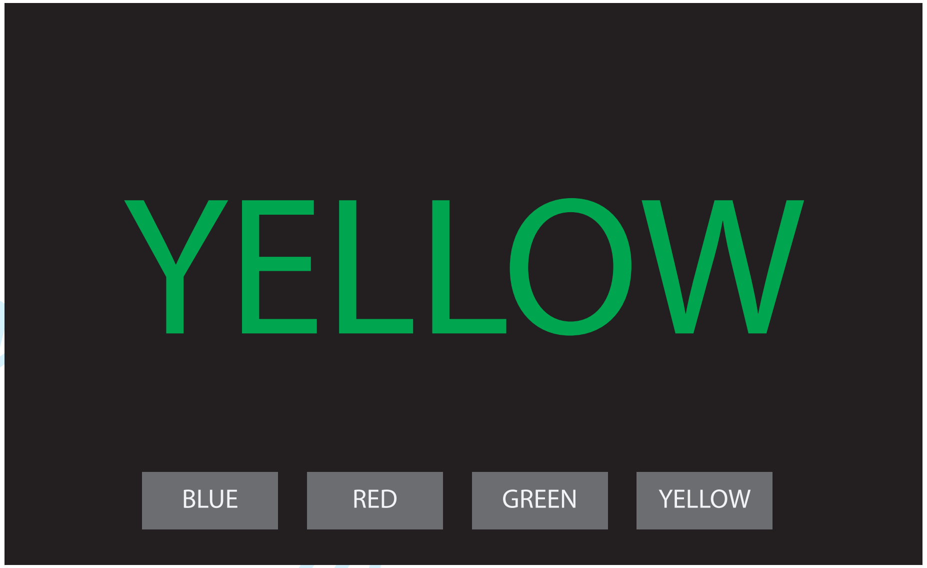

For peer review only - http://bmjopen.bmj.com/site/about/guidelines.xhtml

Patient Reported Outcome Form

1. Are you satisfied with the information you received before the experiment?

Yes ☐ No ☐

If not, could you indicate which kind of information would you have liked to receive regarding the experiment?

____

2. Was the experiment burdensome for you?

Yes ☐ No ☐

If this was the case, could you please specify which part of the experiment was burdensome for you?

____

3. Did you feel uncomfortable during the experiment?

If yes, could you please specify which part of the experiment made you feel uncomfortable?

____

4. Do you consider that the communication during the performance of the experiment and/or about what you could expect during the experiment was adequate?

Yes ☐ No ☐

If this was not the case, could you please specify in which part of the experiment the communication can be improved?

____

5. Did you experience any form of pain during the experiment?

Yes ☐ No ☐

If this was the case, could you please specify in which part of the operation you experienced pain?

____

For peer review only

SPIRIT

STANDARD PROTOCOL ITEMS: RECOMMENDATIONS FOR INTERVENTIONAL TRIALS

SPIRIT 2013 Checklist: Recommended items to address in a clinical trial protocol and related documents*

Section/item	Item No	Description
**Administrative information**		
**Title**	**1**	**Descriptive title identifying the study design, population, interventions, and, if applicable, trial acronym**
**Trial registration**	**2a**	**Trial identifier and registry name. If not yet registered, name of intended registry**
	~~2b~~	~~All items from the World Health Organization Trial Registration Data Set~~
**Protocol version**	**3**	**Date and version identifier**
**Funding**	**4**	**Sources and types of financial, material, and other support**
**Roles and responsibilities**	**5a**	**Names, affiliations, and roles of protocol contributors**
	**5b**	**Name and contact information for the trial sponsor**
	5c	~~Role of study sponsor and funders, if any, in study design; collection, management, analysis, and interpretation of data; writing of the report; and the decision to submit the report for publication, including whether they will have ultimate authority over any of these activities~~
	5d	~~Composition, roles, and responsibilities of the coordinating centre, steering committee, endpoint adjudication committee, data management team, and other individuals or groups overseeing the trial, if applicable (see Item 21a for data monitoring committee)~~
**Introduction**		
**Background and rationale**	**6a**	**Description of research question and justification for undertaking the trial, including summary of relevant studies (published and unpublished) examining benefits and harms for each intervention**
	**6b**	**Explanation for choice of comparators**
**Objectives**	**7**	**Specific objectives or hypotheses**

For peer review only

For peer review only - http://bmjopen.bmj.com/site/about/guidelines.xhtml

**Trial design**	**8**	**Description of trial design including type of trial (eg, parallel group, crossover, factorial, single group), allocation ratio, and framework (eg, superiority, equivalence, noninferiority, exploratory)**

Methods: Participants, interventions, and outcomes

**Study setting**	**9**	**Description of study settings (eg, community clinic, academic hospital) and list of countries where data will be collected. Reference to where list of study sites can be obtained**
**Eligibility criteria**	**10**	**Inclusion and exclusion criteria for participants. If applicable, eligibility criteria for study centres and individuals who will perform the interventions (eg, surgeons, psychotherapists)**
**Interventions**	**11a**	**Interventions for each group with sufficient detail to allow replication, including how and when they will be administered**
	**11b**	**Criteria for discontinuing or modifying allocated interventions for a given trial participant (eg, drug dose change in response to harms, participant request, or improving/worsening disease)**
	**11c**	**Strategies to improve adherence to intervention protocols, and any procedures for monitoring adherence (eg, drug tablet return, laboratory tests)**
	**11d**	**Relevant concomitant care and interventions that are permitted or prohibited during the trial**
**Outcomes**	**12**	**Primary, secondary, and other outcomes, including the specific measurement variable (eg, systolic blood pressure), analysis metric (eg, change from baseline, final value, time to event), method of aggregation (eg, median, proportion), and time point for each outcome. Explanation of the clinical relevance of chosen efficacy and harm outcomes is strongly recommended**
**Participant timeline**	**13**	**Time schedule of enrolment, interventions (including any run-ins and washouts), assessments, and visits for participants. A schematic diagram is highly recommended (see Figure)**
**Sample size**	**14**	**Estimated number of participants needed to achieve study objectives and how it was determined, including clinical and statistical assumptions supporting any sample size calculations**
**Recruitment**	**15**	**Strategies for achieving adequate participant enrolment to reach target sample size**

Methods: Assignment of interventions (for controlled trials)

Allocation:

For peer review only - http://bmjopen.bmj.com/site/about/guidelines.xhtml

**Sequence generation**	**16a**	**Method of generating the allocation sequence (eg, computer-generated random numbers), and list of any factors for stratification. To reduce predictability of a random sequence, details of any planned restriction (eg, blocking) should be provided in a separate document that is unavailable to those who enrol participants or assign interventions**
**Allocation concealment mechanism**	**16b**	**Mechanism of implementing the allocation sequence (eg, central telephone; sequentially numbered, opaque, sealed envelopes), describing any steps to conceal the sequence until interventions are assigned**
~~Implementation~~	~~16c~~	~~Who will generate the allocation sequence, who will enrol participants, and who will assign participants to interventions~~
**Blinding (masking)**	**17a**	**Who will be blinded after assignment to interventions (eg, trial participants, care providers, outcome assessors, data analysts), and how**
	**17b**	**If blinded, circumstances under which unblinding is permissible, and procedure for revealing a participant's allocated intervention during the trial**
**Methods: Data collection, management, and analysis**		
**Data collection methods**	**18a**	**Plans for assessment and collection of outcome, baseline, and other trial data, including any related processes to promote data quality (eg, duplicate measurements, training of assessors) and a description of study instruments (eg, questionnaires, laboratory tests) along with their reliability and validity, if known. Reference to where data collection forms can be found, if not in the protocol**
	~~18b~~	~~Plans to promote participant retention and complete follow-up, including list of any outcome data to be collected for participants who discontinue or deviate from intervention protocols~~
Data management	19	Plans for data entry, coding, security, and storage, including any related processes to promote data quality (eg, double data entry; range checks for data values). Reference to where details of data management procedures can be found, if not in the protocol
**Statistical methods**	**20a**	**Statistical methods for analysing primary and secondary outcomes. Reference to where other details of the statistical analysis plan can be found, if not in the protocol**
	~~20b~~	~~Methods for any additional analyses (eg, subgroup and adjusted analyses)~~
	~~20c~~	~~Definition of analysis population relating to protocol non-adherence (eg, as randomised analysis), and any statistical methods to handle missing data (eg, multiple imputation)~~

For peer review only - http://bmjopen.bmj.com/site/about/guidelines.xhtml

**Methods: Monitoring**		
**Data monitoring**	**21a**	**Composition of data monitoring committee (DMC); summary of its role and reporting structure; statement of whether it is independent from the sponsor and competing interests; and reference to where further details about its charter can be found, if not in the protocol. Alternatively, an explanation of why a DMC is not needed**
	**21b**	**Description of any interim analyses and stopping guidelines, including who will have access to these interim results and make the final decision to terminate the trial**
**Harms**	**22**	**Plans for collecting, assessing, reporting, and managing solicited and spontaneously reported adverse events and other unintended effects of trial interventions or trial conduct**
**Auditing**	**23**	**Frequency and procedures for auditing trial conduct, if any, and whether the process will be independent from investigators and the sponsor**
**Ethics and dissemination**		
**Research ethics approval**	**24**	**Plans for seeking research ethics committee/institutional review board (REC/IRB) approval**
**Protocol amendments**	**25**	**Plans for communicating important protocol modifications (eg, changes to eligibility criteria, outcomes, analyses) to relevant parties (eg, investigators, REC/IRBs, trial participants, trial registries, journals, regulators)**
**Consent or assent**	**26a**	**Who will obtain informed consent or assent from potential trial participants or authorised surrogates, and how (see Item 32)**
	~~26b~~	~~Additional consent provisions for collection and use of participant data and biological specimens in ancillary studies, if applicable~~
**Confidentiality**	**27**	**How personal information about potential and enrolled participants will be collected, shared, and maintained in order to protect confidentiality before, during, and after the trial**
**Declaration of interests**	**28**	**Financial and other competing interests for principal investigators for the overall trial and each study site**
**Access to data**	**29**	**Statement of who will have access to the final trial dataset, and disclosure of contractual agreements that limit such access for investigators**
**Ancillary and post-trial care**	**30**	**Provisions, if any, for ancillary and post-trial care, and for compensation to those who suffer harm from trial participation**

For peer review only

**Dissemination policy**	**31a**	**Plans for investigators and sponsor to communicate trial results to participants, healthcare professionals, the public, and other relevant groups (eg, via publication, reporting in results databases, or other data sharing arrangements), including any publication restrictions**
	~~31b~~	~~Authorship eligibility guidelines and any intended use of professional writers~~
	~~31c~~	~~Plans, if any, for granting public access to the full protocol, participant-level dataset, and statistical code~~
**Appendices**		
~~Informed consent materials~~	~~32~~	~~Model consent form and other related documentation given to participants and authorised surrogates~~
~~Biological specimens~~	~~33~~	~~Plans for collection, laboratory evaluation, and storage of biological specimens for genetic or molecular analysis in the current trial and for future use in ancillary studies, if applicable~~

*It is strongly recommended that this checklist be read in conjunction with the SPIRIT 2013 Explanation & Elaboration for important clarification on the items. Amendments to the protocol should be tracked and dated. The SPIRIT checklist is copyrighted by the SPIRIT Group under the Creative Commons "Attribution-NonCommercial-NoDerivs 3.0 Unported" license.

For peer review only

BMJ Open

Adaptive Deep Brain Stimulation as Advanced Parkinson's disease Treatment (ADAPT study): protocol for a pseudo-randomised clinical study.

Journal:	*BMJ Open*
Manuscript ID	bmjopen-2019-029652.R1
Article Type:	Protocol
Date Submitted by the Author:	06-Mar-2019
Complete List of Authors:	Piña-Fuentes, Dan; University Medical Centre Groningen, Neurosurgery; University Medical Center Groningen<br>Beudel, Martijn; University Medical Center Groningen, Neurology<br>Little, Simon; Institute of Neurology, Queen Square, London, Sobell Department of Motor Neuroscience and Movement Disorders<br>Brown, Peter; University of Oxford, Medical Research Council Brain Network Dynamics Unit<br>Oterdoom, D.L.Marinus; University Medical Center Groningen, Neurosurgery<br>van Dijk, J. Marc C.; University Medical Center Groningen, Neurosurgery
**Primary Subject Heading**:	Neurology
Secondary Subject Heading:	Neurology
Keywords:	Parkinson-s disease < NEUROLOGY, Adaptive Deep Brain Stimulation, beta oscillations, subthalamic nucleus, internal globus pallidus, closed-loop

SCHOLARONE™
Manuscripts

Adaptive Deep Brain Stimulation as Advanced Parkinson's disease Treatment (ADAPT study): protocol for a pseudo-randomised clinical study.

D.A.I. Piña Fuentes MD[1]

M. Beudel MD PhD[2]

Simon Little MBBS, DPhil[3]

P. Brown MD PhD[4]

D. L. M. Oterdoom MD[1]

J.M.C. van Dijk MD PhD[1]

1. Department of Neurosurgery, University Medical Center Groningen, University of Groningen, Groningen, Netherlands

2. Department of Neurology, University Medical Center Groningen, University of Groningen, Groningen, Netherlands

3. Sobell Department of Motor Neuroscience and Movement Disorders, Institute of Neurology, Queen Square, London, UK

4. Medical Research Council Brain Network Dynamics Unit at the University of Oxford, Oxford, UK

Running title: ADAPT study protocol

Corresponding author:

D. Piña-Fuentes MD

Department of Neurosurgery,

University Medical Center Groningen,

Hanzeplein 1,

P. O. Box 30.001,

9700 RB Groningen,

The Netherlands.

tel: +31 50 361 0522

E-mail: d.a.i.pina.fuentes@umcg.nl

Word count

Abstract: 298

Full text: 3813

Number of figures: 4

Number of references: 30

Keywords:

Parkinson's disease, adaptive deep brain stimulation, subthalamic nucleus, internal globus pallidus, local field potentials, beta oscillations, closed-loop.

ABSTRACT

Introduction

Adaptive deep brain stimulation (aDBS), based on the detection of increased beta oscillations in the subthalamic nucleus (STN), has been assessed in Parkinson's disease (PD) patients, during the immediate postoperative setting. In these studies, aDBS was shown to be at least as effective as conventional DBS (cDBS), whilst stimulation time and side effects were reduced. However, the effect of aDBS on motor symptoms and stimulation-induced side effects, during the chronically implanted phase (after the stun effect of DBS placement has disappeared) has not yet been determined.

Methods and analysis

This protocol describes a single-centre clinical study in which aDBS will be tested in 14 PD patients, with electrodes implanted either in the STN or the internal globus pallidus, undergoing battery replacement. Patients included will be allocated in a pseudo-randomised fashion to a 3-condition (no stimulation/ cDBS / aDBS), crossover design. A battery of tests will be conducted and recorded during each condition, which are aimed to measure the severity of motor symptoms and side effects. These tests include a tablet-based tapping test, a subscale of the Movement Disorder Society- unified Parkinson's disease rating scale (subMDS-UPDRS), the Speech Intelligibility Test (SIT) and a tablet-based version of the Stroop Test. SubMDS-UPDRS and SIT recordings will be blindly assessed by independent raters. Data will be analysed using a generalised linear mixed-effects model.

Ethics and dissemination: this protocol was approved by the Ethical Committee of the Institution in which the study will be carried out (University Medical Centre Groningen). Data management and compliance to research policies and standards of our centre, including data privacy, storage and veracity, will be controlled by an independent monitor. All the scientific findings derived from this protocol are aimed to be made public through publication of articles in international journals.

Trial registration number: NTR 5456

Strengths and limitations of this study

This protocol aims to assess both efficacy and side-effect profile of aDBS.

The stun effect will not present in the patient population to be included in this protocol.

Intraoperative testing will minimise exposure time for electrodes and risk of infection.

Testing and washout periods will be limited by the intraoperative time available.

The intraoperative setting will limit the tests available to be assessed in PD patients.

INTRODUCTION

Background and rationale.

Deep brain stimulation (DBS) is currently one of the most effective advanced treatments for patients with Parkinson's disease (PD). At present, conventional DBS (cDBS) provides the continuous delivery of fixed-frequency and -amplitude electrical pulses through the electrodes, implanted mostly in either the subthalamic nucleus (STN) or the internal globus pallidus (GPi).[1,2] Although cDBS is an effective treatment, it still has limitations in terms of efficacy, side effects and efficiency, and these have not undergone substantial improvements since the implementation of cDBS in clinical practice, approximately 20 years ago.[3] However, new DBS-systems directed to improve current stimulation algorithms are currently under development. These systems are often referred to as adaptive DBS

(aDBS), as they aim to modulate the delivery of electrical stimulation, controlled by the detection of symptoms, like tremor, or aberrant signals (biomarkers) that have been implicated in the pathophysiology of the disease.[4] The ultimate goal of aDBS-systems is to reduce the total amount of stimulation delivered, in a way in which beneficial effects of DBS are enhanced, while side effects are reduced.[5] Furthermore, decreasing the total amount of stimulation delivered will allow to extend the battery life(span) of DBS devices.

Several biomarkers in PD have been proposed for use as a feedback signal for aDBS.[6] Currently, the presence of prominent beta oscillations (13-35 Hz), in either the STN or the GPi, seems to be the most promising, since it correlates with the severity of bradykinesia and rigidity.[7] Evidence to date suggests that aDBS may be at least as effective as cDBS systems, whilst stimulation time and side effects are reduced.[8–10] However, virtually all aDBS experiments based on beta oscillations have thus far only been tested in the immediate postoperative setting. Although aDBS has been evaluated mostly with regard to the STN, preliminary evidence indicates that aDBS could be equally effective when applied to the GPi.[11] The correlation of other prominent PD symptoms (such as tremor) with beta oscillations is far less robust. Therefore, the impact of aDBS on these symptoms is yet to be addressed.[12,13]

Besides its effect on motor symptoms, a common side effect that could potentially be addressed by aDBS is stimulation-induced dysarthria. Preliminary data in the immediate postoperative phase showed that aDBS did not induce dysarthria, in contrast to cDBS.[14] Likewise, due to the reduction in the total amount of stimulation that occurs in aDBS, this new technology could potentially help to tackle non-motor side effects induced by stimulation. Certain psychiatric symptoms caused by dopaminergic therapy, such as impulsive behaviour, can be improved by the medication reduction that cDBS allows. On the other hand, stimulation can paradoxically induce impulsive behaviour.[15,16]. It was first proposed that this side effect was due to unintended stimulation of limbic areas of the STN.[17] However, it has been shown recently that DBS has a positive effect on risk-reward decision behaviour in PD patients, and that such an effect was mostly dependent on stimulation of the motor part of the STN.[18] This finding indicates that at least some of the non-motor effects of DBS are mediated through direct stimulation of the motor part of the STN. This is supported by increasing evidence that indicates that the motor and non-motor functions of the STN are not perfectly segregated.[19] Therefore, aDBS might be used as an additional option to address stimulation-induced side effects, such as impulsive behaviour, as it could provide a functional selectivity, by only triggering stimulation based on (primarily) motor biomarkers (e.g. beta oscillations).

Objectives.

The aim of this study is to assess whether aDBS provides a similar motor improvement compared to cDBS, while limiting the presence of stimulation-induced side effects in patients with chronically implanted electrodes. Stimulation efficacy is assessed using a battery of tests, aimed to measure the motor response to different conditions, and the severity of dysarthria and/or impulsivity. The goals of this protocol are summarized in three questions:

I. Is aDBS at least as efficacious as cDBS in terms of motor improvement?

II. Is the speech-related and disinhibition side-effect profile of aDBS lower than that of cDBS?

III. What is the amount of stimulation provided in aDBS compared to cDBS?

Trial design.

This protocol describes an exploratory study in which PD-patients with DBS electrodes implanted on the STN, are allocated to a 3-condition (no stimulation (NoStim) / cDBS / aDBS), crossover design (Figure 1). The conditions are pseudo-randomised in a 2:1:1 format, in such a way that for every 2 patients who start with no-stimulation, 1 patient will receive aDBS first, and 1 will have cDBS first. The main reason for this proposed pseudo-randomisation is to minimise the impact that an incomplete washout-effect derived from stimulation could have on the scores of NoStim condition, as this condition is the baseline for the comparison of the effect of the two stimulation modalities. In four patients, stimulation conditions will be separated by a NoStim condition, to minimize the carryover effect that cDBS could have on aDBS (or vice versa). Lastly, NoStim will be the last condition in two patients, in order to partially balance the effects of training or fatigue on the tasks. Additionally, pilot data could be collected from two patients in order to explore the effects of aDBS on the GPi.

The performance of several tasks during the three conditions will place during the battery replacement surgery, when a temporal externalization of the DBS extension wires is performed. This means that subjects to be included have had their DBS electrodes implanted for at least 3-5 years, and therefore the stun effect is not present in this patient population. It was decided to perform this protocol intraoperatively, as it allows the surgery to be completed in one session, minimising the time that electrodes are externalized and patients are exposed to potential risks of infection.

METHODS AND ANALYSIS:

<u>Methods: Participants, interventions, and outcomes</u>

Study setting.

Single-centre study, at the University Medical Centre Groningen.

Inclusion criteria:

- PD patients with DBS electrodes implanted bilaterally in the STN. A maximum of two PD patients with electrodes implanted in the GPi would be allowed as pilot data.
- Patients are cognitively capable of performing the tests included in this protocol. This item is subjectively evaluated during the patient interview, by looking at the performance of the candidate when completing a trial set of the tablet-based tests used during the experiment.
- Able to provide written informed consent.
- Physical condition that enables 20-30 minutes of testing. This is evaluated according to the clinical records, and the fragility of the patient (determined by the treating neurologist).
- Ability to undergo testing in the OFF dopaminergic state, determined by the clinical condition of the patient after medication withdrawal.

Exclusion criteria:

- Patients who cannot tolerate battery replacement under local anaesthesia (e.g. presence of severe OFF medication tremor, severe anxiety, etc.).
- <18 years old.
- Clinical disorders or diseases that could interfere with clinical assessment (e.g. vision impairment that cannot be corrected with glasses, severe limb movement restriction, etc.).

- Cognitive impairment sufficient to prevent the patient to consider and retain the necessary information to make a decision regarding the participation in the study.

Investigational device.

The responsive (closed loop) aDBS is delivered through a custom-made stimulation-amplifier which allows local field potentials (LFPs) to be recorded during the application of DBS. With this device, LFPs are filtered in real time around a subject specific beta frequency (±3 Hz around peak).[9] Afterwards, a median threshold is selected, in order to deliver monopolar stimulation every time that beta activity in the filtered LFP exceeds the predefined threshold. Stimulation pulses are charge-balanced and asymmetric, mirroring the waveform of the standard clinical Medtronic Activa PC. Pulses are delivered at 130 Hz with a pulse width 60 µs (standard clinical parameters), using a ramping period of 250 ms at onset and offset, in order to help limit the presence of paraesthesias, autonomic or other non-specific side effects, such as dizziness or nausea.[20] From the four contacts available for each electrode lead, 2 electrodes are required to perform bipolar LFP recordings (either contacts 0-2 or 1-3), while one electrode (in between the two recording electrodes) is left available for stimulation (either contacts 1 or 2). The stimulation-amplifier has been built to strict safety guidelines (Report 90 BS EN60601-1 safe design, construction of medical equipment) and extensively tested[8,9,11,21], and found to be safe and reliable.

Procedure.

Patients have to skip at least one medication dose, and are not allowed to be under the effect of prolonged-release dopaminergic medication, in order to be classified as OFF medication before surgery. Patients are brought to the operating theatre for their battery replacement procedure. After the battery is exposed, the old battery is detached from the DBS electrodes and explanted. At this moment, two temporary wires are attached to the DBS electrode extension cables and connected to a combined stimulator and amplifier (See previous section). A brief rest recording of 30-60 seconds is performed, from each of the two bipolar montages (0-2 or 1-3) available for each hemisphere. Power spectral density (PSD) estimates are obtained from each rest recording, and the bipolar contact pair (0-2 or 1-3) per hemisphere with the highest beta peak power is selected. Monopolar cDBS is then titrated, using the available contact located between the selected recording contacts. The voltage for monopolar stimulation is titrated initially based on an approximation of the voltage used in clinical practice for each individual patient (independently of the montage used in clinical practice). Voltage is increased or decreased according to the presence of side effects, clinical effect, and interference with LFP recordings (in practice, we have seen that voltages >3.5 V are more prone to cause interference with the recordings, and these are also rare stimulation parameters in clinical practice). Once an optimum stimulation voltage is established, the patients are assigned to each of 3 counter-balanced conditions in which the patient is either stimulated with aDBS or cDBS, or not stimulated, separated by washout periods of 1-3 minutes, according to the remaining time available. Stimulation parameters (voltage, frequency, pulse width and contact selection) remain constant for both aDBS and cDBS, differing only in the timing of stimulation delivered according to beta detection. As battery replacements are performed under local anaesthesia, we aim to limit the duration of the experiment to a maximum of 25-30 min.

Clinical tests and outcomes:

Each patient performs the following tasks in each of the three pseudo-randomised conditions:

a) Tablet-based tapping test: several analog and digital versions of the tapping test have been validated to objectively measure the presence of bradykinesia in PD patients.[22–24] We developed

a custom tablet-based finger tapping test using the PsyPad platform.[25] In this test, a tablet is presented to the patient, in which two squares appear at the left and right side of the screen (Figure 2). The patient then has to press the squares in an alternating pattern. We tested this setup, as a proof of principle, on a PD patient who underwent battery replacement,[21] using 50 tapping iterations per condition. In order to optimize the time available for the battery of tests, it was decided to reduce the iterations to 20. Since the duration of each iteration was approximately 500ms, 20 iterations (10 back-and-forth screen tapings) will be roughly equivalent to 10 finger tapings, which is the amount of taps required to measure tapping items from the Movement Disorder Society- unified Parkinson's disease rating scale (MDS-UPDRS).

b) Subscale of the MDS-UPDRS (subMDS-UPDRS)[26] (Part III, items 3.4, 3.5, 3.6, 3.15, 3.17 a, b): as patients are lying on the operating table during the experiment, only selected items can be performed by the patients. The items that are included and filmed are: finger tapping, hand movements, pronation-supination, rest tremor, and postural tremor.

c) Speech Intelligibility Test (SIT): this is a modified version of the Assessment of Intelligibility for the Dysarthric Speech.[27,28] In this test, the patient is instructed to read out a list of 18 sentences (approximately 110 words per condition), while the speech is recorded with a mobile app. The words that form each sentence are randomly chosen, taking into account that each sentence must follow the correct order of a proper grammatical construction. Voice recordings are sent to a speech therapist, who blindly evaluates the sentences and writes them down. Afterwards, the sentences written by the speech therapist are compared with the original list, and the amount of words that coincide are expressed as a percentage of the total words.

d) Tablet- based version of the Stroop Test:[29] in this test, 15 colour words are presented to each patient in a sequential order, and each word is coloured in (Figure 3). From those 15 words, 5 are coloured in the same colour as the word written (congruent), while 10 words are coloured in a colour different than the word written (incongruent). We chose an unbalanced design, as our main focus is the effect of stimulation on incongruent words between conditions, rather than the effect of stimulation between congruent and incongruent words. For each word presented, four buttons are presented at the bottom of the tablet screen, each containing different colour words. The patient has to select the button containing the colour word presented on the screen, independently of the colour in which the word is coloured in. Reaction times and accuracy for each trial are directly uploaded to an online database, from which they can be extracted for data analysis.

Order of the tests:

After the battery is exposed, and before the performance of the clinical tests can start, a number of preparations are required in order to program the aDBS (see *Procedure* section). This requirement can lead to individual differences in the time available to perform the clinical tests, due to the maximum amount of intraoperative time allowed for this experiment. Therefore, the sequence in which the clinical tests are performed on each condition is ordered according to the approximate time it takes to perform each of them, the order being as follows: 1. Tabled-based tapping test, 2. subMDS-UPDRS, 3. Speech and Intelligibility Test (SIT), 4. Table-based version of the Stroop Test. If during the course of each individual experiment it is considered that the time will not be sufficient for a patient to complete the full protocol, only the first test(s) could be selected to be performed, in order to ensure that at least one of the tests will be completed for each of the three conditions.

Sample size:

According to previous studies,[30] a difference of ≈4 points in the total motor UPDRS could be considered clinically significant (3.7% of the total scale). This would be roughly equivalent to a limit of 1.5 points on a 10-item UPDRS scale, which is the one used in this protocol. Based on pilot data conducted in Oxford / London on 4 patients,[9] aDBS scores had a standard error of ≈2 points (standard deviation of 4). This is roughly equivalent to a standard deviation of 1.5 points on a 10-item UPDRS scale. Using an α of 0.05, a power (1-β) of 0.8, and assuming similar variances, a sample size of 12 patients would be necessary to demonstrate that the efficacy of aDBS is not inferior to cDBS, while providing the potential benefits of facilitating programming, battery life optimization and the potential to reduce stimulation-induced side effects. A potential extension of 2 patients (14 patients in total) would be pursued, in order to explore the potential effects of aDBS on patients with electrodes implanted on the GPi.

Recruitment:

Patients who are scheduled for a battery replacement at the University Medical Centre Groningen are contacted by telephone 2 weeks prior to their hospital admission to be informed about the study. Complementary written information is sent by mail to patients who show an interest in participating in the study. At the time of hospital admission, each patient is personally approached by one of the members of the research team, if they meet all of the inclusion criteria and none of the exclusion criteria. Patients who decide to participate in the study will then be given the opportunity to ask further questions and invited to sign the consent form.

Methods: Assignment of interventions

Allocation:

The ultimate goal of the allocation procedure is to have a pseudo-randomisation in which all the possible combinations of the block design are covered. (i.e. 3:2:2, see *Trial Design* section, Figure 1). For that reason, patients will be sequentially allocated to each possible pseudo-randomisation scenario, until 14 patients are allocated. Only successful bilateral measurements will be considered in the pseudo-randomisation list. At first, a pseudo-randomisation of 3:1:1 (10 patients) will be set as the allocation goal, in order to ensure that all conditions of the pseudo-randomisation could be met by mid-2019. Depending on the number of aborted experiments and the deadline for inclusion, 4 patients more may potentially be included, to achieve the 3:2:2 pseudo-randomisation.

Blinding (masking)

Patients will be blinded to the stimulation condition. Video and voice recordings will be taken for the subMDS-UPDRS and SIT parts, and will be blindly evaluated by 2 neurologists experienced in movement disorders, and a speech therapist, respectively. Analysis of tablet based outcomes will be automated and matched across the three conditions.

Methods: Data collection, management, and analysis

Data management.

Data is stored on a central database using REDCap storage system, being accessible only to the authorized personnel involved in this study.

Statistical methods.

Simple scores from the subMDS-UPDRS, Tabled-based tapping test, SIT and Stroop Test during aDBS vs cDBS or NoStim will be checked for normality using Q-Q plots, and if normally distributed, will be compared using a generalised linear mixed-effects model. If the data is non-normally distributed a data transformation will be applied, or a distributions different than normal will be employed for the test.

<u>Methods: Monitoring</u>

Harms. The experimental procedure does not substantially expose patients to increased risks, as there are no extra surgical procedures required, and patients are stimulated only with less electrical current than in their conventional setting. Given that the experiments take place in the operation room, and only add less than 30 minutes to the total surgery duration, there is no significant added risk of infection. Since patients are tested in the OFF dopaminergic state, this exaggerates their symptoms to certain extent. However, DBS remains active until the moment of the surgery, which limits the impact of medication discontinuation. In previous aDBS studies, electrodes were exposed for hours or days,[9,10] without any increased risk of infection. Therefore, as battery replacement is completed in one session, the amount of time in which the extension wires are exposed is considerably shortened in this study. Nevertheless, infections will be considered an adverse event and infection rates monitored.

Monitoring. Data management and compliance to research policies and standards of our centre, including data privacy, storage and veracity, are verified by an independent monitor from a different department within the hospital. As this is an investigator-initiated study, the project leader submits, once a year throughout the clinical trial, a safety report to the accredited local ethics committee. Serious adverse events, defined by the Central Committee on Research Involving Human Subjects in the Netherlands as 'any untoward medical occurrence or effect that at any dose results in death, is life threatening (at the time of the event), requires hospitalization or prolongation of existing inpatients' hospitalization or results in persistent or significant disability or incapacity', will be promptly reported to the corresponding governmental authorities.

Patient and Public Involvement. In order to make sure that the experiments are as comfortable as possible for the subjects, a short Patient Reported Outcome (PRO) questionnaire will be conducted on the first five patients (Figure 4). This PRO consists of 5 questions in which patients can indicate whether the communication was adequate and empathetic and whether the procedure was not too demanding, uncomfortable or painful. The feedback of these reports will be used to evaluate the adequacy of the protocol, and changes will be incorporated into the following study procedures, if necessary via an amendment.

ETHICS AND DISSEMINATION:

Research ethics approval.

This protocol is approved by the local ethics committee of our institution, and all procedures are carried out according to the Declaration of Helsinki and the Dutch legislation on medical research involving human subjects.

Protocol amendments.

The protocol presented here is the 5th version (9th December, 2018) of the original protocol prepared for this study. The 2nd version of the protocol (15th September, 2015) got the approval of the ethical committee, after several modifications of the 1st version (22th July, 2015). In version 3 (12th April, 2017), an attempt was made to include patients undergoing new implantations. However, this was dropped in subsequent versions, since: a)patients undergoing new implantations were often considerably fatigued at the moment of the measurement and could not perform all the tasks; b) differences in total intraoperative time between new implantations and battery replacements meant that the time available for the measurement was considerably less during new implantations; c) the stereotactic frame slightly restricted the visual field of the patients in new implantations. Version 4 (7th September, 2017) and version 5 were meant to increase the total number of patients to be included, as several failed or incomplete measurements took place during the initial inclusions.

Consent

During the inclusion interview, patients are informed that the participation in this study is on an entirely voluntary basis, and that they are therefore allowed at any moment to withdraw their consent. No economic reward is provided for the participation in this study.

Confidentiality.

Only indirect personal data from each patient are stored as coded information, in order to prevent the possibility that patient data could be traced back to an individual patient. The coding list will be safeguarded in a secured facility located in our centre. Data derived from the clinical tests or recordings could be exported only as anonymized data to be used for statistical analysis.

Declaration of interests.

All authors declare to have no conflict of interests.

Access to data.

Only authorized personnel have access to the coding list and the software in which the data is stored.

Ancillary and post-trial care

Patients are insured up to € 650,000 for harm suffered directly from trial participation. Details of the insurance are provided on the patient information letter of this study.

Dissemination policy.

All the scientific findings derived from this protocol are aimed to be made public through publication of articles in international journals.

FUNDING STATEMENT: This study is publicly funded by grants received from the Dutch Brain Foundation ('Hersenstichting Nederland', grant F2015(1)-04), the National Mexican Council of Science and Technology (CONACYT) grant number CVU 652927, and the University of Groningen/University Medical Centre Groningen (RuG/UMCG). SL is personally supported by a Wellcome trust post-doctoral clinical research training grant (105804/Z/14/Z)

TRIAL REGISTRATION: NEDERLANDS TRIAL REGISTER – http://www.trialregister.nl

NTR NUMBER: 5456

AUTHOR CONTRIBUTIONS: MB and JMCvD designed and prepared the first draft of the protocol, and initiated the study. DPF formatted the protocol according to the SPIRIT guidelines and prepared the manuscript. SL, PB and DLMO critically reviewed the protocol procedures and manuscript. All authors reviewed and approved the final version of this manuscript.

REFERENCES:

1 Follett KA, Weaver FM, Stern M, *et al.* Pallidal versus subthalamic deep-brain stimulation for Parkinson's disease. *N Engl J Med* 2010;**362**:2077–91. doi:10.1056/NEJMoa0907083

2 Odekerken VJJ, Boel JA, Schmand BA, *et al.* GPi vs STN deep brain stimulation for Parkinson disease: Three-year follow-up. *Neurology* 2016;**86**:755–61. doi:10.1212/WNL.0000000000002401

3 Picillo M, Lozano AM, Kou N, *et al.* Programming Deep Brain Stimulation for Parkinson's Disease: The Toronto Western Hospital Algorithms. *Brain Stimul* 2016;**9**:425–37. doi:10.1016/J.BRS.2016.02.004

4 Priori A, Foffani G, Rossi L, *et al.* Adaptive deep brain stimulation (aDBS) controlled by local field potential oscillations. *Exp Neurol* 2013;**245**:77–86. doi:10.1016/J.EXPNEUROL.2012.09.013

5 Beudel M, Brown P. Adaptive deep brain stimulation in Parkinson's disease. *Parkinsonism Relat Disord* 2016;**22 Suppl 1**:S123-6. doi:10.1016/j.parkreldis.2015.09.028

6 Hoang KB, Cassar IR, Grill WM, *et al.* Biomarkers and Stimulation Algorithms for Adaptive Brain Stimulation. *Front Neurosci* 2017;**11**:564. doi:10.3389/fnins.2017.00564

7 Neumann W-J, Degen K, Schneider G-H, *et al.* Subthalamic synchronized oscillatory activity correlates with motor impairment in patients with Parkinson's disease. *Mov Disord* 2016;**31**:1748–51. doi:10.1002/mds.26759

8 Little S, Pogosyan A, Neal S, *et al.* Adaptive Deep Brain Stimulation In Advanced Parkinson Disease. Ann. Neurol. 2013;**74**:449–57. doi:10.1002/ana.23951

9 Little S, Beudel M, Zrinzo L, *et al.* Bilateral adaptive deep brain stimulation is effective in Parkinson's disease. *J Neurol Neurosurg Psychiatry* 2016;**87**:717–21. doi:10.1136/jnnp-2015-310972

10 Arlotti M, Marceglia S, Foffani G, *et al.* Eight-hours adaptive deep brain stimulation in patients with Parkinson disease. *Neurology* 2018;**90**:e971–6. doi:10.1212/WNL.0000000000005121

11 Pina-Fuentes D, van Zijl JC, van Dijk JMC, *et al.* The characteristics of pallidal low-frequency and beta bursts could help implementing adaptive brain stimulation in the parkinsonian and dystonic internal globus pallidus. *Neurobiol Dis* 2019;**121**:47–57. doi:10.1016/j.nbd.2018.09.014

12 Qasim SE, de Hemptinne C, Swann NC, *et al.* Electrocorticography reveals beta desynchronization in the basal ganglia-cortical loop during rest tremor in Parkinson's disease. *Neurobiol Dis* 2016;**86**:177–86. doi:10.1016/j.nbd.2015.11.023

13 Beudel M, Oswal A, Jha A, *et al.* Oscillatory Beta Power Correlates With Akinesia-Rigidity in the Parkinsonian Subthalamic Nucleus. Mov. Disord. 2017;**32**:174–5. doi:10.1002/mds.26860

14 Little S, Tripoliti E, Beudel M, *et al.* Adaptive deep brain stimulation for Parkinson's disease demonstrates reduced speech side effects compared to conventional stimulation in the acute setting. J. Neurol. Neurosurg. Psychiatry. 2016;**87**:1388–9. doi:10.1136/jnnp-2016-313518

15 Kim A, Kim YE, Kim H-J, *et al.* A 7-year observation of the effect of subthalamic deep brain stimulation on impulse control disorder in patients with Parkinson's disease. *Parkinsonism Relat Disord* Published Online First: 20 July 2018. doi:10.1016/J.PARKRELDIS.2018.07.010

16 Abbes M, Lhommee E, Thobois S, *et al.* Subthalamic stimulation and neuropsychiatric symptoms in Parkinson's disease: results from a long-term follow-up cohort study. *J Neurol Neurosurg Psychiatry* 2018;**89**:836–43. doi:10.1136/jnnp-2017-316373

17 Winter C, Lemke C, Sohr R, *et al.* High frequency stimulation of the subthalamic nucleus modulates neurotransmission in limbic brain regions of the rat. *Exp brain Res* 2008;**185**:497–507. doi:10.1007/s00221-007-1171-1

18 Irmen F, Horn A, Meder D, *et al.* Sensorimotor subthalamic stimulation restores risk-reward trade-off in Parkinson's disease. *Mov Disord* 2018;:1–11. doi:10.1002/mds.27576

19 Eisenstein SA, Koller JM, Black KD, *et al.* Functional anatomy of subthalamic nucleus stimulation in Parkinson disease. *Ann Neurol* 2014;**76**:279–95. doi:10.1002/ana.24204

20 Dayal V, Limousin P, Foltynie T. Subthalamic Nucleus Deep Brain Stimulation in Parkinson's Disease: The Effect of Varying Stimulation Parameters. *J Parkinsons Dis* 2017;**7**:235–45. doi:10.3233/JPD-171077

21 Piña-Fuentes D, Little S, Oterdoom M, *et al.* Adaptive DBS in a Parkinson's patient with chronically implanted DBS: A proof of principle. *Mov Disord* 2017;**32**. doi:10.1002/mds.26959

22 Bronte-stewart HM, Ding L, Alexander C, *et al.* Quantitative Digitography (QDG): A Sensitive Measure of Digital Motor Control in Idiopathic Parkinson ' s Disease. 2000;**15**:36–47.

23 Noyce AJ, Treacy C, Budu C, *et al.* The new Bradykinesia Akinesia Incoordination (BRAIN) test: preliminary data from an online test of upper limb movement. Mov. Disord. 2012;**27**:157–8. doi:10.1002/mds.23947

24 Kassavetis P, Saifee TA, Roussos G, *et al.* Developing a Tool for Remote Digital Assessment of Parkinson's Disease. *Mov Disord Clin Pract* 2016;**3**:59–64. doi:10.1002/mdc3.12239

25 Turpin A, Lawson DJ, McKendrick AM. PsyPad: a platform for visual psychophysics on the iPad. *J Vis* 2014;**14**:16. doi:10.1167/14.3.16

26 Goetz CG, Tilley BC, Shaftman SR, *et al.* Movement Disorder Society-sponsored revision of the Unified Parkinson's Disease Rating Scale (MDS-UPDRS): scale presentation and clinimetric testing results. *Mov Disord* 2008;**23**:2129–70. doi:10.1002/mds.22340

27 Yorkston KM, Beukelman DR. Communication efficiency of dysarthric speakers as measured by sentence intelligibility and speaking rate. *J Speech Hear Disord* 1981;**46**:296–301.

28 Tripoliti E, Zrinzo L, Martinez-Torres I, *et al.* Effects of contact location and voltage amplitude on speech and movement in bilateral subthalamic nucleus deep brain stimulation. *Mov Disord* 2008;**23**:2377–83. doi:10.1002/mds.22296

29 Stroop JR. Studies of interference in serial verbal reactions. *J Exp Psychol* 1935;**18**:643.

30 Hauser RA, Auinger P. Determination of minimal clinically important change in early and advanced Parkinson's disease. *Mov Disord* 2011;**26**:813–8. doi:10.1002/mds.23638

Figure 1. Trial design and pseudo-randomisation block.

Figure 2. Example of a trial of the tablet-based bradykinesia test.

Figure 3. Example of two trials of the tablet-based Stroop test with congruent (left) and incongruent (right) conditions.

Figure 4. Example of the Patient Reported Outcome questionnaire form conducted on the participants.

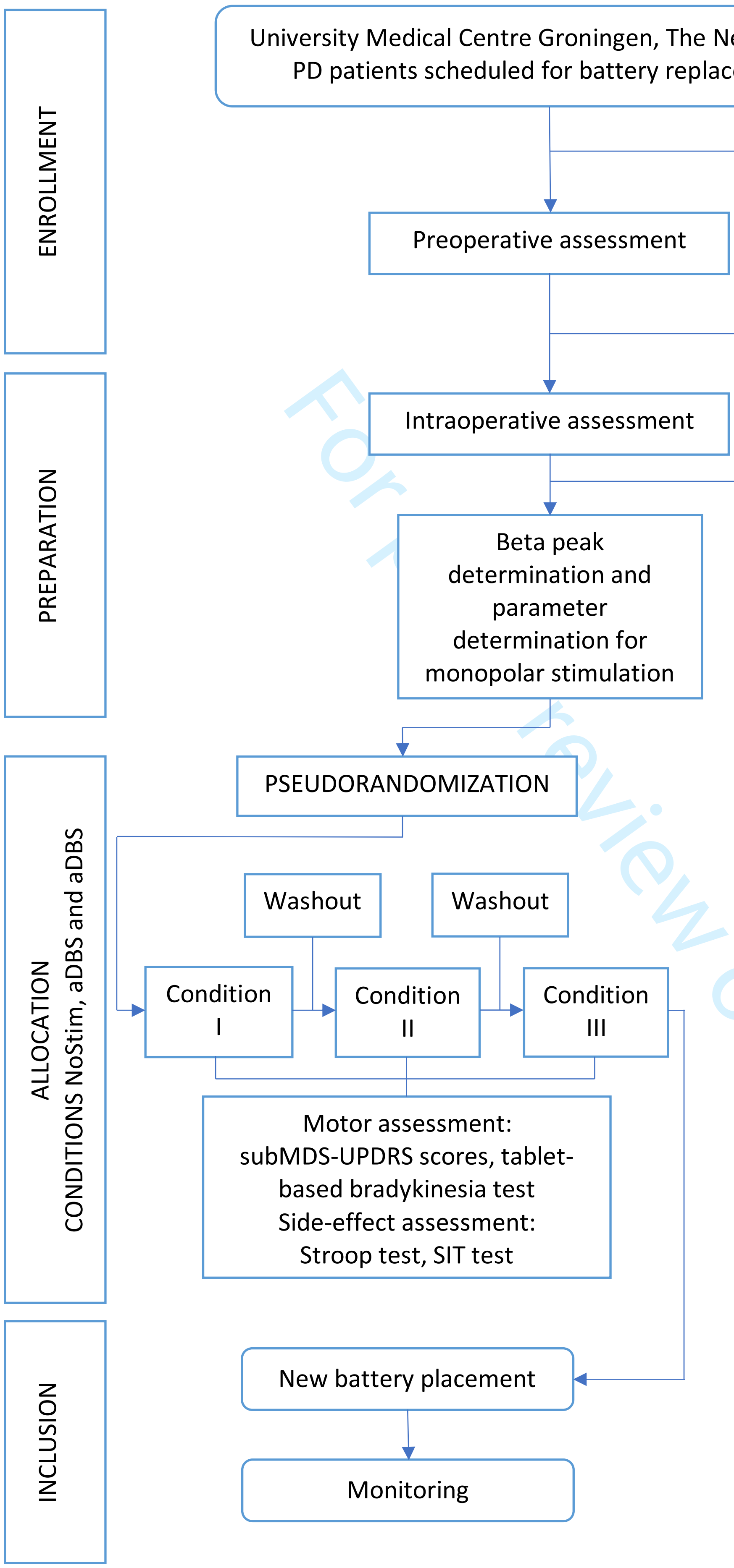

PSEUDORANDOMIZED BLOCK DESIGN		
Condition I	Condition II	Condition III
NoStim	cDBS	aDBS
NoStim	cDBS	aDBS
NoStim	cDBS	aDBS
NoStim	aDBS	cDBS
NoStim	aDBS	cDBS
NoStim	aDBS	cDBS
cDBS	NoStim	aDBS
cDBS	NoStim	aDBS
cDBS	aDBS	NoStim
aDBS	NoStim	cDBS
aDBS	NoStim	cDBS
aDBS	cDBS	NoStim
	**GPi pilot data**	
NoStim	aDBS	cDBS
NoStim	cDBS	aDBS

For peer review only - http://bmjopen.bmj.com/site/about/guidelines.xhtml

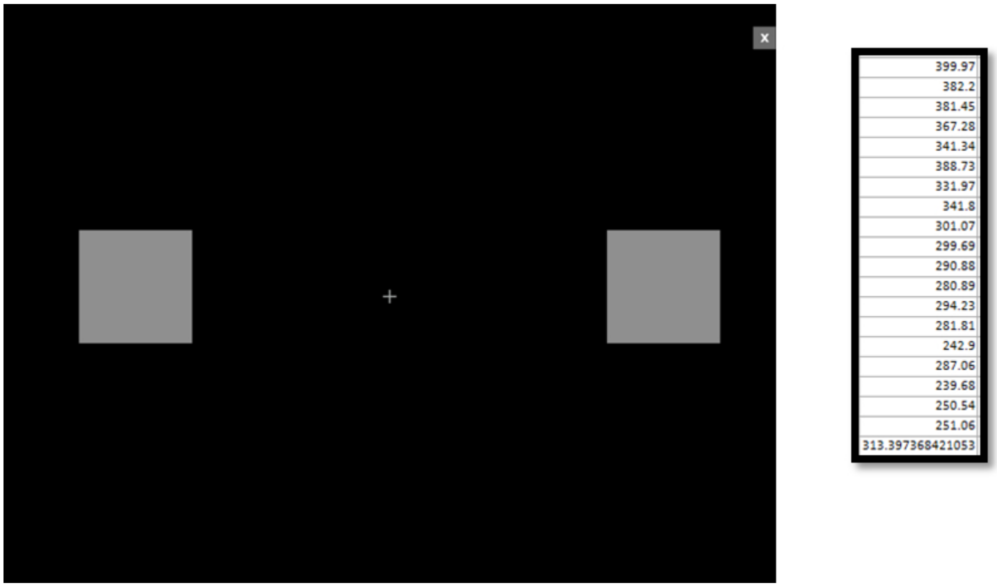

Figure 2. Example of a trial of the tablet-based bradykinesia test.

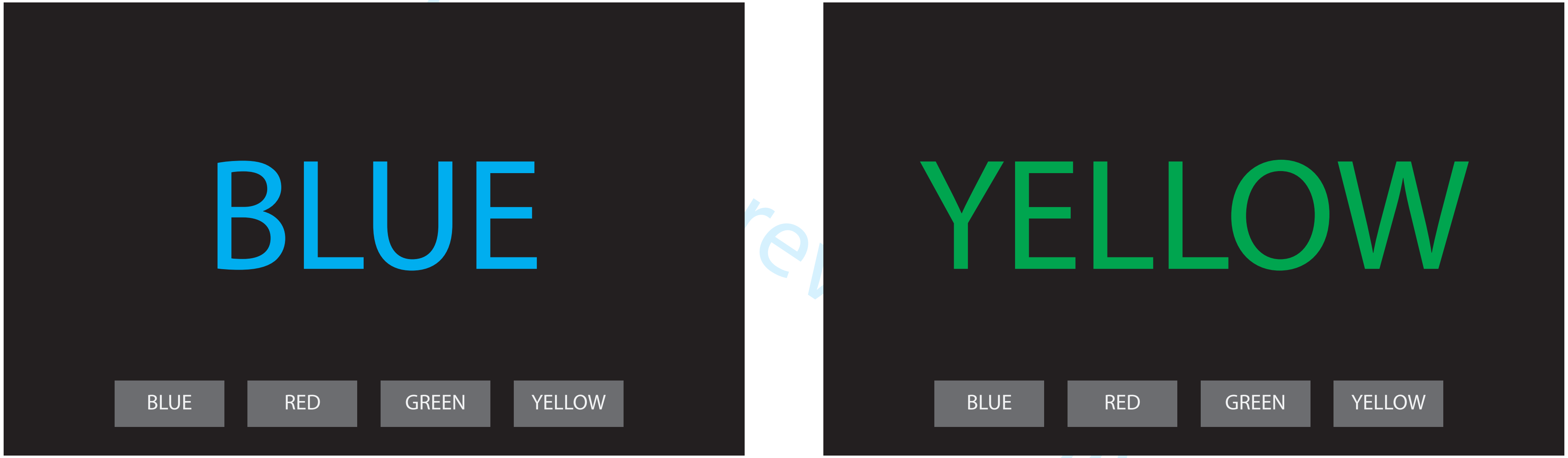
BLUE
BLUE
RED
GREEN
YELLOW
YELLOW
BLUE
RED
GREEN
YELLOW

Patient Reported Outcome Form

1. Are you satisfied with the information you received before the experiment?

Yes ☐ No ☐

If not, could you indicate which kind of information would you have liked to receive regarding the experiment?

____

____

2. Was the experiment burdensome for you?

Yes ☐ No ☐

If this was the case, could you please specify which part of the experiment was burdensome for you?

____

____

3. Did you feel uncomfortable during the experiment?

If yes, could you please specify which part of the experiment made you feel uncomfortable?

____

____

4. Do you consider that the communication during the performance of the experiment and/or about what you could expect during the experiment was adequate?

Yes ☐ No ☐

If this was not the case, could you please specify in which part of the experiment the communication can be improved?

____

____

5. Did you experience any form of pain during the experiment?

Yes ☐ No ☐

If this was the case, could you please specify in which part of the operation you experienced pain?

____

____

For peer review only

SPIRIT

STANDARD PROTOCOL ITEMS: RECOMMENDATIONS FOR INTERVENTIONAL TRIALS

SPIRIT 2013 Checklist: Recommended items to address in a clinical trial protocol and related documents*

Section/item	Item No	Description
**Administrative information**		
**Title**<br>**Page 1, Line 3**	**1**	**Descriptive title identifying the study design, population, interventions, and, if applicable, trial acronym**
**Trial registration**<br>**Page 2, Line 35**	**2a**	**Trial identifier and registry name. If not yet registered, name of intended registry**
	~~2b~~	~~All items from the World Health Organization Trial Registration Data Set~~
**Protocol version**<br>**Page 9, Line 16**	**3**	**Date and version identifier**
**Funding**<br>**Page 10, Line 6**	**4**	**Sources and types of financial, material, and other support**
**Roles and responsibilities**<br>**Page 10, Line 18**	**5a**	**Names, affiliations, and roles of protocol contributors**
	**5b**	**Name and contact information for the trial sponsor**
	5c	~~Role of study sponsor and funders, if any, in study design; collection, management, analysis, and interpretation of data; writing of the report; and the decision to submit the report for publication, including whether they will have ultimate authority over any of these activities~~
	5d	~~Composition, roles, and responsibilities of the coordinating centre, steering committee, endpoint adjudication committee, data management team, and other individuals or groups overseeing the trial, if applicable (see Item 21a for data monitoring committee)~~
**Introduction**		
**Background and rationale**<br>**Page 2, Line 55**	**6a**	**Description of research question and justification for undertaking the trial, including summary of relevant studies (published and unpublished) examining benefits and harms for each intervention**
	**6b**	**Explanation for choice of comparators**
**Objectives**<br>**Page 3, Line 52**	**7**	**Specific objectives or hypotheses**

For peer review only

**Trial design Page 4, Line 10**	**8**	**Description of trial design including type of trial (eg, parallel group, crossover, factorial, single group), allocation ratio, and framework (eg, superiority, equivalence, noninferiority, exploratory)**

Methods: Participants, interventions, and outcomes

**Study setting Page 4, Line 39**	**9**	**Description of study settings (eg, community clinic, academic hospital) and list of countries where data will be collected. Reference to where list of study sites can be obtained**
**Eligibility criteria Page 4, Line 42**	**10**	**Inclusion and exclusion criteria for participants. If applicable, eligibility criteria for study centres and individuals who will perform the interventions (eg, surgeons, psychotherapists)**
**Interventions Page 5, Line 36**	**11a**	**Interventions for each group with sufficient detail to allow replication, including how and when they will be administered**
	**11b**	**Criteria for discontinuing or modifying allocated interventions for a given trial participant (eg, drug dose change in response to harms, participant request, or improving/worsening disease)**
	**11c**	**Strategies to improve adherence to intervention protocols, and any procedures for monitoring adherence (eg, drug tablet return, laboratory tests)**
	**11d**	**Relevant concomitant care and interventions that are permitted or prohibited during the trial**
**Outcomes Page 6, Line 8**	**12**	**Primary, secondary, and other outcomes, including the specific measurement variable (eg, systolic blood pressure), analysis metric (eg, change from baseline, final value, time to event), method of aggregation (eg, median, proportion), and time point for each outcome. Explanation of the clinical relevance of chosen efficacy and harm outcomes is strongly recommended**
**Participant timeline Page 5, Line 36. Figure 1**	**13**	**Time schedule of enrolment, interventions (including any run-ins and washouts), assessments, and visits for participants. A schematic diagram is highly recommended (see Figure)**
**Sample size Page 7, Line 11**	**14**	**Estimated number of participants needed to achieve study objectives and how it was determined, including clinical and statistical assumptions supporting any sample size calculations**
**Recruitment Page 7, Line 33**	**15**	**Strategies for achieving adequate participant enrolment to reach target sample size**

Methods: Assignment of interventions (for controlled trials)

Allocation:

For peer review only - http://bmjopen.bmj.com/site/about/guidelines.xhtml

**Sequence generation Figure 1**	**16a**	**Method of generating the allocation sequence (eg, computer-generated random numbers), and list of any factors for stratification. To reduce predictability of a random sequence, details of any planned restriction (eg, blocking) should be provided in a separate document that is unavailable to those who enrol participants or assign interventions**
**Allocation concealment mechanism Page 7, Line 48**	**16b**	**Mechanism of implementing the allocation sequence (eg, central telephone; sequentially numbered, opaque, sealed envelopes), describing any steps to conceal the sequence until interventions are assigned**
~~Implementation~~	~~16c~~	~~Who will generate the allocation sequence, who will enrol participants, and who will assign participants to interventions~~
**Blinding (masking) Page 8, Line 3**	**17a**	**Who will be blinded after assignment to interventions (eg, trial participants, care providers, outcome assessors, data analysts), and how**
	**17b**	**If blinded, circumstances under which unblinding is permissible, and procedure for revealing a participant's allocated intervention during the trial**
**Methods: Data collection, management, and analysis**		
**Data collection methods Page 5, Line 16 Page 5, Line 35**	**18a**	**Plans for assessment and collection of outcome, baseline, and other trial data, including any related processes to promote data quality (eg, duplicate measurements, training of assessors) and a description of study instruments (eg, questionnaires, laboratory tests) along with their reliability and validity, if known. Reference to where data collection forms can be found, if not in the protocol**
	~~18b~~	~~Plans to promote participant retention and complete follow-up, including list of any outcome data to be collected for participants who discontinue or deviate from intervention protocols~~
Data management **Page 8, Line 12**	19	Plans for data entry, coding, security, and storage, including any related processes to promote data quality (eg, double data entry; range checks for data values). Reference to where details of data management procedures can be found, if not in the protocol
**Statistical methods Page 8, Line 17**	**20a**	**Statistical methods for analysing primary and secondary outcomes. Reference to where other details of the statistical analysis plan can be found, if not in the protocol**
	~~20b~~	~~Methods for any additional analyses (eg, subgroup and adjusted analyses)~~
	~~20c~~	~~Definition of analysis population relating to protocol non-adherence (eg, as randomised analysis), and any statistical methods to handle missing data (eg, multiple imputation)~~

For peer review only - http://bmjopen.bmj.com/site/about/guidelines.xhtml

Methods: Monitoring

**Data monitoring<br>Page 8, Line 44**	**21a**	**Composition of data monitoring committee (DMC); summary of its role and reporting structure; statement of whether it is independent from the sponsor and competing interests; and reference to where further details about its charter can be found, if not in the protocol. Alternatively, an explanation of why a DMC is not needed**
	**21b**	**Description of any interim analyses and stopping guidelines, including who will have access to these interim results and make the final decision to terminate the trial**
**Harms<br>Page 8, Line 30**	**22**	**Plans for collecting, assessing, reporting, and managing solicited and spontaneously reported adverse events and other unintended effects of trial interventions or trial conduct**
**Auditing<br>Page 8, Line 44**	**23**	**Frequency and procedures for auditing trial conduct, if any, and whether the process will be independent from investigators and the sponsor**

Ethics and dissemination

**Research ethics approval<br>Page 9, Line 10**	**24**	**Plans for seeking research ethics committee/institutional review board (REC/IRB) approval**
**Protocol amendments<br>Page 9 , Line 16**	**25**	**Plans for communicating important protocol modifications (eg, changes to eligibility criteria, outcomes, analyses) to relevant parties (eg, investigators, REC/IRBs, trial participants, trial registries, journals, regulators)**
**Consent or assent<br>Page 9, Line 32**	**26a**	**Who will obtain informed consent or assent from potential trial participants or authorised surrogates, and how (see Item 32)**
	~~26b~~	~~Additional consent provisions for collection and use of participant data and biological specimens in ancillary studies, if applicable~~
**Confidentiality<br>Page 9, Line 38**	**27**	**How personal information about potential and enrolled participants will be collected, shared, and maintained in order to protect confidentiality before, during, and after the trial**
**Declaration of interests<br>Page 9, Line 46**	**28**	**Financial and other competing interests for principal investigators for the overall trial and each study site**
**Access to data<br>Page 9, Line 50**	**29**	**Statement of who will have access to the final trial dataset, and disclosure of contractual agreements that limit such access for investigators**

For peer review only

**Ancillary and post-trial care Page 9, Line 25**	**30**	**Provisions, if any, for ancillary and post-trial care, and for compensation to those who suffer harm from trial participation**
**Dissemination policy Page 9, Line 53**	**31a**	**Plans for investigators and sponsor to communicate trial results to participants, healthcare professionals, the public, and other relevant groups (eg, via publication, reporting in results databases, or other data sharing arrangements), including any publication restrictions**
	~~31b~~	~~Authorship eligibility guidelines and any intended use of professional writers~~
	~~31c~~	~~Plans, if any, for granting public access to the full protocol, participant-level dataset, and statistical code~~
**Appendices**		
~~Informed consent materials~~	~~32~~	~~Model consent form and other related documentation given to participants and authorised surrogates~~
~~Biological specimens~~	~~33~~	~~Plans for collection, laboratory evaluation, and storage of biological specimens for genetic or molecular analysis in the current trial and for future use in ancillary studies, if applicable~~

*It is strongly recommended that this checklist be read in conjunction with the SPIRIT 2013 Explanation & Elaboration for important clarification on the items. Amendments to the protocol should be tracked and dated. The SPIRIT checklist is copyrighted by the SPIRIT Group under the Creative Commons "Attribution-NonCommercial-NoDerivs 3.0 Unported" license.

For peer review only

BMJ Open

Adaptive Deep Brain Stimulation as Advanced Parkinson's disease Treatment (ADAPT study): protocol for a pseudo-randomised clinical study.

Journal:	*BMJ Open*
Manuscript ID	bmjopen-2019-029652.R2
Article Type:	Protocol
Date Submitted by the Author:	01-Apr-2019

Complete List of Authors:	Piña-Fuentes, Dan; University Medical Centre Groningen, Neurosurgery; University Medical Center Groningen<br>Beudel, Martijn; University Medical Center Groningen, Neurology<br>Little, Simon; Institute of Neurology, Queen Square, London, Sobell Department of Motor Neuroscience and Movement Disorders<br>Brown, Peter; University of Oxford, Medical Research Council Brain Network Dynamics Unit<br>Oterdoom, D.L.Marinus; University Medical Center Groningen, Neurosurgery<br>van Dijk, J. Marc C.; University Medical Center Groningen, Neurosurgery

**Primary Subject Heading**:	Neurology
Secondary Subject Heading:	Neurology
Keywords:	Parkinson-s disease < NEUROLOGY, Adaptive Deep Brain Stimulation, beta oscillations, subthalamic nucleus, internal globus pallidus, closed-loop

SCHOLARONE™ Manuscripts

Adaptive Deep Brain Stimulation as Advanced Parkinson's disease Treatment (ADAPT study): protocol for a pseudo-randomised clinical study.

D.A.I. Piña Fuentes MD[1]

M. Beudel MD PhD[2]

Simon Little MBBS, DPhil[3]

P. Brown MD PhD[4]

D. L. M. Oterdoom MD[1]

J.M.C. van Dijk MD PhD[1]

1. Department of Neurosurgery, University Medical Center Groningen, University of Groningen, Groningen, Netherlands

2. Department of Neurology, University Medical Center Groningen, University of Groningen, Groningen, Netherlands

3. Sobell Department of Motor Neuroscience and Movement Disorders, Institute of Neurology, Queen Square, London, UK

4. Medical Research Council Brain Network Dynamics Unit at the University of Oxford, Oxford, UK

Running title: ADAPT study protocol

Corresponding author:

D. Piña-Fuentes MD

Department of Neurosurgery,

University Medical Center Groningen,

Hanzeplein 1,

P. O. Box 30.001,

9700 RB Groningen,

The Netherlands.

tel: +31 50 361 0522

E-mail: d.a.i.pina.fuentes@umcg.nl

Word count

Abstract: 300

Full text: 3929

Number of figures: 4

Number of references: 35

Keywords:

Parkinson's disease, adaptive deep brain stimulation, subthalamic nucleus, internal globus pallidus, local field potentials, beta oscillations, closed-loop.

ABSTRACT

Introduction

Adaptive deep brain stimulation (aDBS), based on the detection of increased beta oscillations in the subthalamic nucleus (STN), has been assessed in Parkinson's disease (PD) patients, during the immediate postoperative setting. In these studies, aDBS was shown to be at least as effective as conventional DBS (cDBS), whilst stimulation time and side effects were reduced. However, the effect of aDBS on motor symptoms and stimulation-induced side effects, during the chronically implanted phase (after the stun effect of DBS placement has disappeared) has not yet been determined.

Methods and analysis

This protocol describes a single-centre clinical study in which aDBS will be tested in 12 PD patients undergoing battery replacement, with electrodes implanted in the STN, and a proof of concept in the internal globus pallidus. Patients included will be allocated in a pseudo-randomised fashion to a 3-condition (no stimulation/ cDBS / aDBS), crossover design. A battery of tests will be conducted and recorded during each condition, which are aimed to measure the severity of motor symptoms and side effects. These tests include a tablet-based tapping test, a subscale of the Movement Disorder Society- unified Parkinson's disease rating scale (subMDS-UPDRS), the Speech Intelligibility Test (SIT) and a tablet- based version of the Stroop Test. SubMDS-UPDRS and SIT recordings will be blindly assessed by independent raters. Data will be analysed using a generalised linear mixed-effects model.

Ethics and dissemination: this protocol was approved by the Ethical Committee of the University Medical Centre Groningen, where the study will be carried out. Data management and compliance to research policies and standards of our centre, including data privacy, storage and veracity, will be controlled by an independent monitor. All the scientific findings derived from this protocol are aimed to be made public through publication of articles in international journals.

Trial registration number: NTR 5456

Strengths and limitations of this study

This protocol aims to assess both efficacy and side-effect profile of aDBS.

The stun effect will not present in the patient population to be included in this protocol.

Intraoperative testing will minimise exposure time for electrodes and risk of infection.

Testing and washout periods will be limited by the intraoperative time available.

The intraoperative setting will limit the tests available to be assessed in PD patients.

INTRODUCTION

Background and rationale.

Deep brain stimulation (DBS) is currently one of the most effective advanced treatments for patients with Parkinson's disease (PD). At present, conventional DBS (cDBS) provides the continuous delivery of fixed-frequency and -amplitude electrical pulses through the electrodes, implanted mostly in either the subthalamic nucleus (STN) or the internal globus pallidus (GPi).[1,2] Although cDBS is an effective treatment, it still has limitations in terms of efficacy, side effects and efficiency, and these have not undergone substantial improvements since the implementation of cDBS in clinical practice, approximately 20 years ago.[3] However, new DBS-systems directed to improve current stimulation

algorithms are currently under development. These systems are often referred to as adaptive DBS (aDBS), as they aim to modulate the delivery of electrical stimulation, controlled by the detection of symptoms, like tremor, or aberrant signals (biomarkers) that have been implicated in the pathophysiology of the disease.[4] The ultimate goal of aDBS-systems is to reduce the total amount of stimulation delivered, in a way in which beneficial effects of DBS are enhanced, while side effects are reduced.[5] Furthermore, decreasing the total amount of stimulation delivered will allow to extend the battery life(span) of DBS devices.

Several biomarkers in PD have been proposed for use as a feedback signal for aDBS.[6] Currently, the presence of prominent beta oscillations (13-35 Hz), in either the STN or the GPi, seems to be the most promising, since it correlates with the severity of bradykinesia and rigidity.[7] Evidence to date suggests that aDBS may be at least as effective as cDBS systems, whilst stimulation time and side effects are reduced.[8–10] However, virtually all aDBS experiments based on beta oscillations have thus far only been tested in the immediate postoperative setting. Although aDBS has been evaluated mostly with regard to the STN, preliminary evidence indicates that aDBS could be equally effective when applied to the GPi.[11] The correlation of other prominent PD symptoms (such as tremor) with beta oscillations is far less robust. Therefore, the impact of aDBS on these symptoms is yet to be addressed.[12,13]

Besides its effect on motor symptoms, a common side effect that could potentially be addressed by aDBS is stimulation-induced dysarthria. Preliminary data in the immediate postoperative phase showed that aDBS did not induce dysarthria, in contrast to cDBS.[14] Likewise, due to the reduction in the total amount of stimulation that occurs in aDBS, this new technology could potentially help to tackle DBS-related side effects in executive functions, such as impaired response inhibition.[15,16] PD patients treated with DBS are prone to make more mistakes in inhibitory control tests, e.g. the Stroop task.[17] Response inhibition is a fundamental cognitive function, and its impairment could lead to impulsive conduct.[18] For that reason, it is possible that stimulation-related impulsivity is the result of an impairment of response inhibition caused by DBS.[19,20] It was first proposed that this side effect was due to unintended stimulation of limbic areas of the STN.[21] However, it has been shown recently that DBS has a positive effect on risk-reward decision behaviour in PD patients, and that such an effect was mostly dependent on stimulation of the motor part of the STN.[22] This finding indicates that at least some of the non-motor effects of DBS are mediated through direct stimulation of the motor part of the STN. This is supported by increasing evidence that indicates that the motor and non-motor functions of the STN are not perfectly segregated.[23] It has been shown that beta oscillations in the motor STN play a significant role in an adequate response inhibition.[24] For that reason, stimulation-induced impulsivity could be the result of an excessive beta suppression produced by continuous stimulation. Therefore, aDBS might be used as an additional option to address stimulation-induced impulsivity, since aDBS has the potential to provide a functional selectivity, by only triggering stimulation based on (primarily) motor biomarkers (e.g. beta oscillations), only when a threshold is exceeded. As a result of it, only excessive beta activity would be suppressed.

Objectives.

The aim of this study is to assess whether aDBS provides a similar motor improvement compared to cDBS, while limiting the presence of stimulation-induced side effects in patients with chronically implanted electrodes. Stimulation efficacy is assessed using a battery of tests, aimed to measure the motor response to different conditions, and the severity of dysarthria and/or impulsivity. The goals of this protocol are summarized in three questions:

I. Is aDBS at least as efficacious as cDBS in terms of motor improvement?

II. Is the speech-related and response inhibition side-effect profile of aDBS lower than that of cDBS?

III. What is the amount of stimulation provided in aDBS compared to cDBS?

Trial design.

This protocol describes an exploratory study in which PD-patients with DBS electrodes implanted on the STN, are allocated to a 3-condition (no stimulation (NoStim) / cDBS / aDBS), crossover design (Figure 1). The conditions are pseudo-randomised in a 2:1:1 format, in such a way that for every 2 patients who start with no-stimulation, 1 patient will receive aDBS first, and 1 will have cDBS first. The main reason for this proposed pseudo-randomisation is to minimise the impact that an incomplete washout-effect derived from stimulation could have on the scores of NoStim condition, as this condition is the baseline for the comparison of the effect of the two stimulation modalities. In four patients, stimulation conditions will be separated by a NoStim condition, to minimize the carryover effect that cDBS could have on aDBS (or vice versa). Lastly, NoStim will be the last condition in two patients, in order to partially balance the effects of training or fatigue on the tasks. Additionally, pilot data could be collected from two patients in order to explore the effects of aDBS on the GPi.

The performance of several tasks during the three conditions will place during the battery replacement surgery, when a temporal externalization of the DBS extension wires is performed. This means that subjects to be included have had their DBS electrodes implanted for at least 3-5 years, and therefore the stun effect is not present in this patient population. It was decided to perform this protocol intraoperatively, as it allows the surgery to be completed in one session, minimising the time that electrodes are externalized and patients are exposed to potential risks of infection.

METHODS AND ANALYSIS:

Methods: Participants, interventions, and outcomes

Study setting.

Single-centre study, at the University Medical Centre Groningen.

Inclusion criteria:

- PD patients with DBS electrodes implanted bilaterally in the STN. A maximum of two PD patients with electrodes implanted in the GPi would be allowed as pilot data.
- Patients are cognitively capable of performing the tests included in this protocol. This item is subjectively evaluated during the patient interview, by looking at the performance of the candidate when completing a trial set of the tablet-based tests used during the experiment.
- Able to provide written informed consent.
- Physical condition that enables 20-30 minutes of testing. This is evaluated according to the clinical records, and the fragility of the patient (determined by the treating neurologist).
- Ability to undergo testing in the OFF dopaminergic state, determined by the clinical condition of the patient after medication withdrawal.

Exclusion criteria:

- Patients who cannot tolerate battery replacement under local anaesthesia (e.g. presence of severe OFF medication tremor, severe anxiety, etc.).
- <18 years old.
- Clinical disorders or diseases that could interfere with clinical assessment (e.g. vision impairment that cannot be corrected with glasses, severe limb movement restriction, etc.).
- Cognitive impairment sufficient to prevent the patient to consider and retain the necessary information to make a decision regarding the participation in the study.

Investigational device.

The responsive (closed loop) aDBS is delivered through a custom-made stimulation-amplifier which allows local field potentials (LFPs) to be recorded during the application of DBS. With this device, LFPs are filtered in real time around a subject specific beta frequency (±3 Hz around peak).[9] Afterwards, a median threshold is selected, in order to deliver monopolar stimulation every time that beta activity in the filtered LFP exceeds the predefined threshold. Stimulation pulses are charge-balanced and asymmetric, mirroring the waveform of the standard clinical Medtronic Activa PC. Pulses are delivered at 130 Hz with a pulse width 60 µs (standard clinical parameters), using a ramping period of 250 ms at onset and offset, in order to help limit the presence of paraesthesias, autonomic or other non-specific side effects, such as dizziness or nausea.[25] From the four contacts available for each electrode lead, 2 electrodes are required to perform bipolar LFP recordings (either contacts 0-2 or 1-3), while one electrode (in between the two recording electrodes) is left available for stimulation (either contacts 1 or 2). The stimulation-amplifier has been built to strict safety guidelines (Report 90 BS EN60601-1 safe design, construction of medical equipment) and extensively tested[8,9,11,26], and found to be safe and reliable.

Procedure.

Patients have to skip at least one medication dose, and are not allowed to be under the effect of prolonged-release dopaminergic medication, in order to be classified as OFF medication before surgery. Patients are brought to the operating theatre for their battery replacement procedure. After the battery is exposed, the old battery is detached from the DBS electrodes and explanted. At this moment, two temporary wires are attached to the DBS electrode extension cables and connected to a combined stimulator and amplifier (See previous section). A brief rest recording of 30-60 seconds is performed, from each of the two bipolar montages (0-2 or 1-3) available for each hemisphere. Power spectral density (PSD) estimates are obtained from each rest recording, and the bipolar contact pair (0-2 or 1-3) per hemisphere with the highest beta peak power is selected. Monopolar cDBS is then titrated, using the available contact located between the selected recording contacts. The voltage for monopolar stimulation is titrated initially based on an approximation of the voltage used in clinical practice for each individual patient (independently of the montage used in clinical practice). Voltage is increased or decreased according to the presence of side effects, clinical effect, and interference with LFP recordings (in practice, we have seen that voltages >3.5 V are more prone to cause interference with the recordings, and these are also rare stimulation parameters in clinical practice). Once an optimum stimulation voltage is established, the patients are assigned to each of 3 counter-balanced conditions in which the patient is either stimulated with aDBS or cDBS, or not stimulated, separated by washout periods of 1-3 minutes, according to the remaining time available. Stimulation parameters (voltage, frequency, pulse width and contact selection) remain constant for both aDBS and cDBS, differing only in the timing of stimulation delivered according to beta detection. As battery

replacements are performed under local anaesthesia, we aim to limit the duration of the experiment to a maximum of 25-30 min.

Clinical tests and outcomes:

Each patient performs the following tasks in each of the three pseudo-randomised conditions:

a) Tablet-based tapping test: several analog and digital versions of the tapping test have been validated to objectively measure the presence of bradykinesia in PD patients.[27–29] We developed a custom tablet-based finger tapping test using the PsyPad platform.[30] In this test, a tablet is presented to the patient, in which two squares appear at the left and right side of the screen (Figure 2). The patient then has to press the squares in an alternating pattern. We tested this setup, as a proof of principle, on a PD patient who underwent battery replacement,[26] using 50 tapping iterations per condition. In order to optimize the time available for the battery of tests, it was decided to reduce the iterations to 20. Since the duration of each iteration was approximately 500ms, 20 iterations (10 back-and-forth screen tapings) will be roughly equivalent to 10 finger tapings, which is the amount of taps required to measure tapping items from the Movement Disorder Society- unified Parkinson's disease rating scale (MDS-UPDRS).

b) Subscale of the MDS-UPDRS (subMDS-UPDRS)[31] (Part III, items 3.4, 3.5, 3.6, 3.15, 3.17 a, b): as patients are lying on the operating table during the experiment, only selected items can be performed by the patients. The items that are included and filmed are: finger tapping, hand movements, pronation-supination, rest tremor, and postural tremor.

c) Speech Intelligibility Test (SIT): this is a modified version of the Assessment of Intelligibility for the Dysarthric Speech.[32,33] In this test, the patient is instructed to read out a list of 18 sentences (approximately 110 words per condition), while the speech is recorded with a mobile app. The words that form each sentence are randomly chosen, taking into account that each sentence must follow the correct order of a proper grammatical construction. Voice recordings are sent to a speech therapist, who blindly evaluates the sentences and writes them down. Afterwards, the sentences written by the speech therapist are compared with the original list, and the amount of words that coincide are expressed as a percentage of the total words.

d) Tablet- based version of the Stroop Test:[34] in this test, 15 colour words are presented to each patient in a sequential order, and each word is coloured in (Figure 3). From those 15 words, 5 are coloured in the same colour as the word written (congruent), while 10 words are coloured in a colour different than the word written (incongruent). We chose an unbalanced design, as our main focus is the effect of stimulation on incongruent words between conditions, rather than the effect of stimulation between congruent and incongruent words. For each word presented, four buttons are presented at the bottom of the tablet screen, each containing different colour words. The patient has to select the button containing the colour word presented on the screen, independently of the colour in which the word is coloured in. Reaction times and accuracy for each trial are directly uploaded to an online database, from which they can be extracted for data analysis.

Order of the tests:

After the battery is exposed, and before the performance of the clinical tests can start, a number of preparations are required in order to program the aDBS (see *Procedure* section). This requirement can lead to individual differences in the time available to perform the clinical tests, due to the maximum amount of intraoperative time allowed for this experiment. Therefore, the sequence in which the clinical tests are performed on each condition is ordered according to the approximate

time it takes to perform each of them, the order being as follows: 1. Tabled-based tapping test, 2. subMDS-UPDRS, 3. Speech and Intelligibility Test (SIT), 4. Table-based version of the Stroop Test. If during the course of each individual experiment it is considered that the time will not be sufficient for a patient to complete the full protocol, only the first test(s) could be selected to be performed, in order to ensure that at least one of the tests will be completed for each of the three conditions.

Sample size:

According to previous studies,[35] a difference of ≈4 points in the total motor UPDRS could be considered clinically significant (3.7% of the total scale). This would be roughly equivalent to a limit of 1.5 points on a 10-item UPDRS scale, which is the one used in this protocol. Based on pilot data conducted in Oxford / London on 4 patients,[9] aDBS scores had a standard error of ≈2 points (standard deviation of 4). This is roughly equivalent to a standard deviation of 1.5 points on a 10-item UPDRS scale. Using an α of 0.05, a power (1-β) of 0.8, and assuming similar variances, a sample size of 12 patients would be necessary to demonstrate that the efficacy of aDBS is not inferior to cDBS, while providing the potential benefits of facilitating programming, battery life optimization and the potential to reduce stimulation-induced side effects. A potential extension of 2 patients (14 patients in total) would be pursued, in order to explore the potential effects of aDBS on patients with electrodes implanted on the GPi.

Recruitment:

Patients who are scheduled for a battery replacement at the University Medical Centre Groningen are contacted by telephone 2 weeks prior to their hospital admission to be informed about the study. Complementary written information is sent by mail to patients who show an interest in participating in the study. At the time of hospital admission, each patient is personally approached by one of the members of the research team, if they meet all of the inclusion criteria and none of the exclusion criteria. Patients who decide to participate in the study will then be given the opportunity to ask further questions and invited to sign the consent form.

Methods: Assignment of interventions

Allocation:

The ultimate goal of the allocation procedure is to have a pseudo-randomisation in which all the possible combinations of the block design are covered. (i.e. 2:1:1, see *Trial Design* section, Figure 1). For that reason, patients will be sequentially allocated to each possible pseudo-randomisation scenario, until 14 patients are allocated. Only successful bilateral measurements will be considered in the pseudo-randomisation list. At first, a pseudo-randomisation of 3:1:1 (10 patients) will be set as the allocation goal, in order to ensure that all conditions of the pseudo-randomisation could be met by mid-2019. Depending on the number of aborted experiments and the deadline for inclusion, 2 patients more may potentially be included, to achieve the 2:1:1 pseudo-randomisation.

Blinding (masking)

Patients will be blinded to the stimulation condition. Video and voice recordings will be taken for the subMDS-UPDRS and SIT parts, and will be blindly evaluated by 2 neurologists experienced in movement disorders, and a speech therapist, respectively. Analysis of tablet based outcomes will be automated and matched across the three conditions.

Methods: Data collection, management, and analysis

Data management.

Data is stored on a central database using REDCap storage system, being accessible only to the authorized personnel involved in this study.

Statistical methods.

Simple scores from the subMDS-UPDRS, Tabled-based tapping test, SIT and Stroop Test during aDBS vs cDBS or NoStim will be checked for normality using Q-Q plots, and if normally distributed, will be compared using a generalised linear mixed-effects model. If the data is non-normally distributed a data transformation will be applied, or a distributions different than normal will be employed for the test.

Methods: Monitoring

Harms. The experimental procedure does not substantially expose patients to increased risks, as there are no extra surgical procedures required, and patients are stimulated only with less electrical current than in their conventional setting. Given that the experiments take place in the operation room, and only add less than 30 minutes to the total surgery duration, there is no significant added risk of infection. Since patients are tested in the OFF dopaminergic state, this exaggerates their symptoms to certain extent. However, DBS remains active until the moment of the surgery, which limits the impact of medication discontinuation. In previous aDBS studies, electrodes were exposed for hours or days,[9,10] without any increased risk of infection. Therefore, as battery replacement is completed in one session, the amount of time in which the extension wires are exposed is considerably shortened in this study. Nevertheless, infections will be considered an adverse event and infection rates monitored.

Monitoring. Data management and compliance to research policies and standards of our centre, including data privacy, storage and veracity, are verified by an independent monitor from a different department within the hospital. As this is an investigator-initiated study, the project leader submits, once a year throughout the clinical trial, a safety report to the accredited local ethics committee. Serious adverse events, defined by the Central Committee on Research Involving Human Subjects in the Netherlands as 'any untoward medical occurrence or effect that at any dose results in death, is life threatening (at the time of the event), requires hospitalization or prolongation of existing inpatients' hospitalization or results in persistent or significant disability or incapacity', will be promptly reported to the corresponding governmental authorities.

Patient and Public Involvement. In order to make sure that the experiments are as comfortable as possible for the subjects, a short Patient Reported Outcome (PRO) questionnaire will be conducted on the first five patients (Figure 4). This PRO consists of 5 questions in which patients can indicate whether the communication was adequate and empathetic and whether the procedure was not too demanding, uncomfortable or painful. The feedback of these reports will be used to evaluate the adequacy of the protocol, and changes will be incorporated into the following study procedures, if necessary via an amendment.

ETHICS AND DISSEMINATION:

Research ethics approval.

This protocol is approved by the local ethics committee of our institution, and all procedures are carried out according to the Declaration of Helsinki and the Dutch legislation on medical research involving human subjects.

Protocol amendments.

The protocol presented here is the 5th version (9th December, 2018) of the original protocol prepared for this study. The 2nd version of the protocol (15th September, 2015) got the approval of the ethical committee, after several modifications of the 1st version (22th July, 2015). In version 3 (12th April, 2017), an attempt was made to include patients undergoing new implantations. However, this was dropped in subsequent versions, since: a)patients undergoing new implantations were often considerably fatigued at the moment of the measurement and could not perform all the tasks; b) differences in total intraoperative time between new implantations and battery replacements meant that the time available for the measurement was considerably less during new implantations; c) the stereotactic frame slightly restricted the visual field of the patients in new implantations. Version 4 (7th September, 2017) and version 5 were meant to increase the total number of patients to be included, as several failed or incomplete measurements took place during the initial inclusions.

Consent

During the inclusion interview, patients are informed that the participation in this study is on an entirely voluntary basis, and that they are therefore allowed at any moment to withdraw their consent. No economic reward is provided for the participation in this study.

Confidentiality.

Only indirect personal data from each patient are stored as coded information, in order to prevent the possibility that patient data could be traced back to an individual patient. The coding list will be safeguarded in a secured facility located in our centre. Data derived from the clinical tests or recordings could be exported only as anonymized data to be used for statistical analysis.

Declaration of interests.

All authors declare to have no conflict of interests.

Access to data.

Only authorized personnel have access to the coding list and the software in which the data is stored.

Ancillary and post-trial care

Patients are insured up to € 650,000 for harm suffered directly from trial participation. Details of the insurance are provided on the patient information letter of this study.

Dissemination policy.

All the scientific findings derived from this protocol are aimed to be made public through publication of articles in international journals.

FUNDING STATEMENT: This study is publicly funded by grants received from the Dutch Brain Foundation ('Hersenstichting Nederland', grant F2015(1)-04), the National Mexican Council of Science and Technology (CONACYT) grant number CVU 652927, and the University of

Groningen/University Medical Centre Groningen (RuG/UMCG). SL is personally supported by a Wellcome trust post-doctoral clinical research training grant (105804/Z/14/Z)

TRIAL REGISTRATION: NEDERLANDS TRIAL REGISTER – http://www.trialregister.nl

NTR NUMBER: 5456

AUTHOR CONTRIBUTIONS: MB and JMCvD designed and prepared the first draft of the protocol, and initiated the study. DPF formatted the protocol according to the SPIRIT guidelines and prepared the manuscript. SL, PB and DLMO critically reviewed the protocol procedures and manuscript. All authors reviewed and approved the final version of this manuscript.

REFERENCES:

1 Follett KA, Weaver FM, Stern M, *et al.* Pallidal versus subthalamic deep-brain stimulation for Parkinson's disease. *N Engl J Med* 2010;**362**:2077–91. doi:10.1056/NEJMoa0907083

2 Odekerken VJJ, Boel JA, Schmand BA, *et al.* GPi vs STN deep brain stimulation for Parkinson disease: Three-year follow-up. *Neurology* 2016;**86**:755–61. doi:10.1212/WNL.0000000000002401

3 Picillo M, Lozano AM, Kou N, *et al.* Programming Deep Brain Stimulation for Parkinson's Disease: The Toronto Western Hospital Algorithms. *Brain Stimul* 2016;**9**:425–37. doi:10.1016/J.BRS.2016.02.004

4 Priori A, Foffani G, Rossi L, *et al.* Adaptive deep brain stimulation (aDBS) controlled by local field potential oscillations. *Exp Neurol* 2013;**245**:77–86. doi:10.1016/J.EXPNEUROL.2012.09.013

5 Beudel M, Brown P. Adaptive deep brain stimulation in Parkinson's disease. *Parkinsonism Relat Disord* 2016;**22 Suppl 1**:S123-6. doi:10.1016/j.parkreldis.2015.09.028

6 Hoang KB, Cassar IR, Grill WM, *et al.* Biomarkers and Stimulation Algorithms for Adaptive Brain Stimulation. *Front Neurosci* 2017;**11**:564. doi:10.3389/fnins.2017.00564

7 Neumann W-J, Degen K, Schneider G-H, *et al.* Subthalamic synchronized oscillatory activity correlates with motor impairment in patients with Parkinson's disease. *Mov Disord* 2016;**31**:1748–51. doi:10.1002/mds.26759

8 Little S, Pogosyan A, Neal S, *et al.* Adaptive deep brain stimulation in advanced Parkinson disease. *Ann Neurol* 2013;**74**:449–57. doi:10.1002/ana.23951

9 Little S, Beudel M, Zrinzo L, *et al.* Bilateral adaptive deep brain stimulation is effective in Parkinson's disease. *J Neurol Neurosurg Psychiatry* 2016;**87**:717–21. doi:10.1136/jnnp-2015-310972

10 Arlotti M, Marceglia S, Foffani G, *et al.* Eight-hours adaptive deep brain stimulation in patients with Parkinson disease. *Neurology* 2018;**90**:e971–6. doi:10.1212/WNL.0000000000005121

11 Pina-Fuentes D, van Zijl JC, van Dijk JMC, *et al.* The characteristics of pallidal low-frequency and beta bursts could help implementing adaptive brain stimulation in the parkinsonian and dystonic internal globus pallidus. *Neurobiol Dis* 2019;**121**:47–57. doi:10.1016/j.nbd.2018.09.014

12 Qasim SE, de Hemptinne C, Swann NC, *et al.* Electrocorticography reveals beta desynchronization in the basal ganglia-cortical loop during rest tremor in Parkinson's disease.

Neurobiol Dis 2016;**86**:177–86. doi:10.1016/j.nbd.2015.11.023

13 Beudel M, Oswal A, Jha A, *et al.* Oscillatory Beta Power Correlates With Akinesia-Rigidity in the Parkinsonian Subthalamic Nucleus. Mov. Disord. 2017;**32**:174–5. doi:10.1002/mds.26860

14 Little S, Tripoliti E, Beudel M, *et al.* Adaptive deep brain stimulation for Parkinson's disease demonstrates reduced speech side effects compared to conventional stimulation in the acute setting. J. Neurol. Neurosurg. Psychiatry. 2016;**87**:1388–9. doi:10.1136/jnnp-2016-313518

15 Frank MJ, Samanta J, Moustafa AA, *et al.* Hold your horses: impulsivity, deep brain stimulation, and medication in parkinsonism. *Science* 2007;**318**:1309–12. doi:10.1126/science.1146157

16 Ray NJ, Jenkinson N, Brittain J, *et al.* The role of the subthalamic nucleus in response inhibition: evidence from deep brain stimulation for Parkinson's disease. *Neuropsychologia* 2009;**47**:2828–34. doi:10.1016/j.neuropsychologia.2009.06.011

17 Witt K, Pulkowski U, Herzog J, *et al.* Deep brain stimulation of the subthalamic nucleus improves cognitive flexibility but impairs response inhibition in Parkinson disease. *Arch Neurol* 2004;**61**:697–700. doi:10.1001/archneur.61.5.697

18 Bari A, Robbins TW. Inhibition and impulsivity: behavioral and neural basis of response control. *Prog Neurobiol* 2013;**108**:44–79. doi:10.1016/j.pneurobio.2013.06.005

19 Kim A, Kim YE, Kim H-J, *et al.* A 7-year observation of the effect of subthalamic deep brain stimulation on impulse control disorder in patients with Parkinson's disease. *Parkinsonism Relat Disord* Published Online First: 20 July 2018. doi:10.1016/J.PARKRELDIS.2018.07.010

20 Abbes M, Lhommee E, Thobois S, *et al.* Subthalamic stimulation and neuropsychiatric symptoms in Parkinson's disease: results from a long-term follow-up cohort study. *J Neurol Neurosurg Psychiatry* 2018;**89**:836–43. doi:10.1136/jnnp-2017-316373

21 Winter C, Lemke C, Sohr R, *et al.* High frequency stimulation of the subthalamic nucleus modulates neurotransmission in limbic brain regions of the rat. *Exp brain Res* 2008;**185**:497–507. doi:10.1007/s00221-007-1171-1

22 Irmen F, Horn A, Meder D, *et al.* Sensorimotor subthalamic stimulation restores risk-reward trade-off in Parkinson's disease. *Mov Disord* 2018;:1–11. doi:10.1002/mds.27576

23 Eisenstein SA, Koller JM, Black KD, *et al.* Functional anatomy of subthalamic nucleus stimulation in Parkinson disease. *Ann Neurol* 2014;**76**:279–95. doi:10.1002/ana.24204

24 Brittain J-S, Watkins KE, Joundi RA, *et al.* A role for the subthalamic nucleus in response inhibition during conflict. *J Neurosci* 2012;**32**:13396–401. doi:10.1523/JNEUROSCI.2259-12.2012

25 Dayal V, Limousin P, Foltynie T. Subthalamic Nucleus Deep Brain Stimulation in Parkinson's Disease: The Effect of Varying Stimulation Parameters. *J Parkinsons Dis* 2017;**7**:235–45. doi:10.3233/JPD-171077

26 Piña-Fuentes D, Little S, Oterdoom M, *et al.* Adaptive DBS in a Parkinson's patient with chronically implanted DBS: A proof of principle. *Mov Disord* 2017;**32**. doi:10.1002/mds.26959

27 Bronte-stewart HM, Ding L, Alexander C, *et al.* Quantitative Digitography (QDG): A Sensitive Measure of Digital Motor Control in Idiopathic Parkinson ' s Disease. 2000;**15**:36–47.

28 Noyce AJ, Treacy C, Budu C, *et al.* The new Bradykinesia Akinesia Incoordination (BRAIN) test: preliminary data from an online test of upper limb movement. Mov. Disord. 2012;**27**:157–8.

doi:10.1002/mds.23947

29 Kassavetis P, Saifee TA, Roussos G, *et al.* Developing a Tool for Remote Digital Assessment of Parkinson's Disease. *Mov Disord Clin Pract* 2016;**3**:59–64. doi:10.1002/mdc3.12239

30 Turpin A, Lawson DJ, McKendrick AM. PsyPad: a platform for visual psychophysics on the iPad. *J Vis* 2014;**14**:16. doi:10.1167/14.3.16

31 Goetz CG, Tilley BC, Shaftman SR, *et al.* Movement Disorder Society-sponsored revision of the Unified Parkinson's Disease Rating Scale (MDS-UPDRS): scale presentation and clinimetric testing results. *Mov Disord* 2008;**23**:2129–70. doi:10.1002/mds.22340

32 Yorkston KM, Beukelman DR. Communication efficiency of dysarthric speakers as measured by sentence intelligibility and speaking rate. *J Speech Hear Disord* 1981;**46**:296–301.

33 Tripoliti E, Zrinzo L, Martinez-Torres I, *et al.* Effects of contact location and voltage amplitude on speech and movement in bilateral subthalamic nucleus deep brain stimulation. *Mov Disord* 2008;**23**:2377–83. doi:10.1002/mds.22296

34 Stroop JR. Studies of interference in serial verbal reactions. *J Exp Psychol* 1935;**18**:643.

35 Hauser RA, Auinger P. Determination of minimal clinically important change in early and advanced Parkinson's disease. *Mov Disord* 2011;**26**:813–8. doi:10.1002/mds.23638

Figure 1. Trial design and pseudo-randomisation block.

Figure 2. Example of a trial of the tablet-based bradykinesia test.

Figure 3. Example of two trials of the tablet-based Stroop test with congruent (left) and incongruent (right) conditions.

Figure 4. Example of the Patient Reported Outcome questionnaire form conducted on the participants.

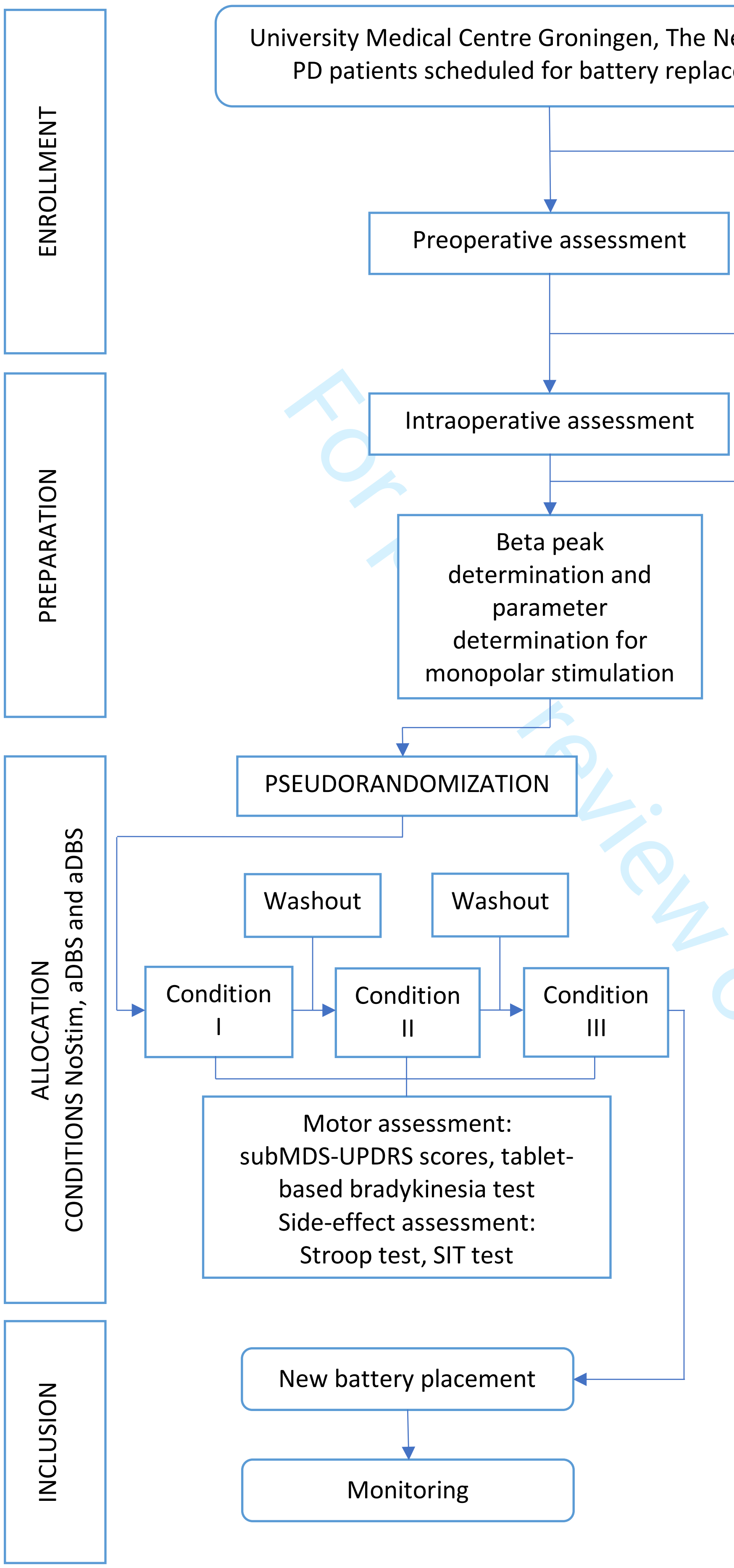

PSEUDORANDOMIZED BLOCK DESIGN		
Condition I	Condition II	Condition III
NoStim	cDBS	aDBS
NoStim	cDBS	aDBS
NoStim	cDBS	aDBS
NoStim	aDBS	cDBS
NoStim	aDBS	cDBS
NoStim	aDBS	cDBS
cDBS	NoStim	aDBS
cDBS	NoStim	aDBS
cDBS	aDBS	NoStim
aDBS	NoStim	cDBS
aDBS	NoStim	cDBS
aDBS	cDBS	NoStim
	**GPi pilot data**	
NoStim	aDBS	cDBS
NoStim	cDBS	aDBS

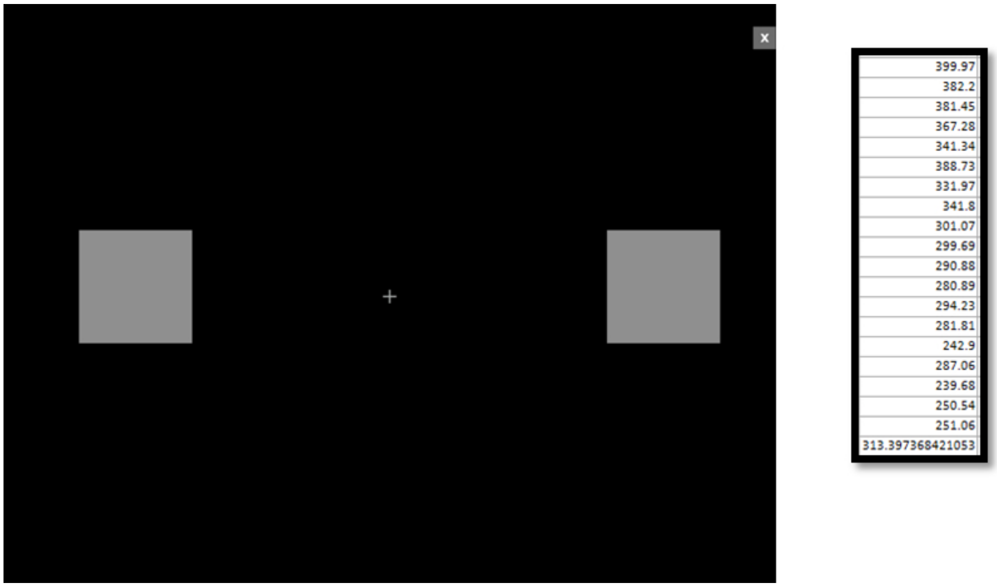

Figure 2. Example of a trial of the tablet-based bradykinesia test.

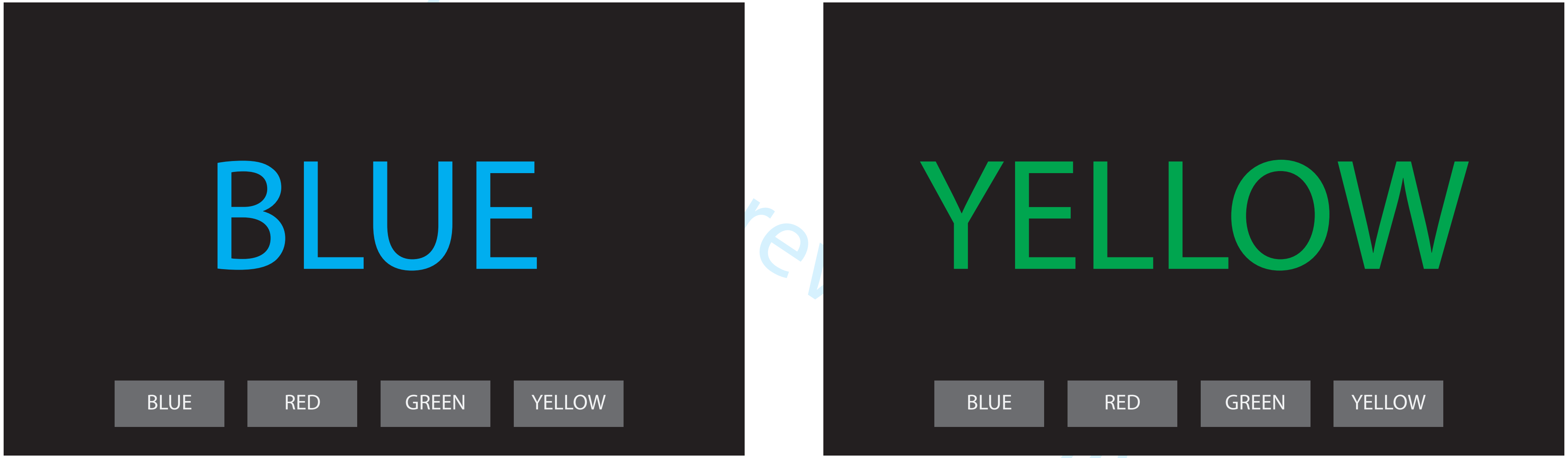

For peer review only - http://bmjopen.bmj.com/site/about/guidelines.xhtml

Patient Reported Outcome Form

1. Are you satisfied with the information you received before the experiment?

Yes ☐ No ☐

If not, could you indicate which kind of information would you have liked to receive regarding the experiment?

___

___

2. Was the experiment burdensome for you?

Yes ☐ No ☐

If this was the case, could you please specify which part of the experiment was burdensome for you?

___

___

3. Did you feel uncomfortable during the experiment?

If yes, could you please specify which part of the experiment made you feel uncomfortable?

___

___

4. Do you consider that the communication during the performance of the experiment and/or about what you could expect during the experiment was adequate?

Yes ☐ No ☐

If this was not the case, could you please specify in which part of the experiment the communication can be improved?

___

___

5. Did you experience any form of pain during the experiment?

Yes ☐ No ☐

If this was the case, could you please specify in which part of the operation you experienced pain?

___

___

For peer review only

SPIRIT

STANDARD PROTOCOL ITEMS: RECOMMENDATIONS FOR INTERVENTIONAL TRIALS

SPIRIT 2013 Checklist: Recommended items to address in a clinical trial protocol and related documents*

Section/item	Item No	Description
**Administrative information**		
**Title**<br>**Page 1, Line 3**	**1**	**Descriptive title identifying the study design, population, interventions, and, if applicable, trial acronym**
**Trial registration**<br>**Page 2, Line 35**	**2a**	**Trial identifier and registry name. If not yet registered, name of intended registry**
	~~2b~~	~~All items from the World Health Organization Trial Registration Data Set~~
**Protocol version**<br>**Page 9, Line 16**	**3**	**Date and version identifier**
**Funding**<br>**Page 10, Line 6**	**4**	**Sources and types of financial, material, and other support**
**Roles and responsibilities**<br>**Page 10, Line 18**	**5a**	**Names, affiliations, and roles of protocol contributors**
	**5b**	**Name and contact information for the trial sponsor**
	5c	~~Role of study sponsor and funders, if any, in study design; collection, management, analysis, and interpretation of data; writing of the report; and the decision to submit the report for publication, including whether they will have ultimate authority over any of these activities~~
	5d	~~Composition, roles, and responsibilities of the coordinating centre, steering committee, endpoint adjudication committee, data management team, and other individuals or groups overseeing the trial, if applicable (see Item 21a for data monitoring committee)~~
**Introduction**		
**Background and rationale**<br>**Page 2, Line 55**	**6a**	**Description of research question and justification for undertaking the trial, including summary of relevant studies (published and unpublished) examining benefits and harms for each intervention**
	**6b**	**Explanation for choice of comparators**
**Objectives**<br>**Page 3, Line 52**	**7**	**Specific objectives or hypotheses**

For peer review only

**Trial design Page 4, Line 10**	**8**	**Description of trial design including type of trial (eg, parallel group, crossover, factorial, single group), allocation ratio, and framework (eg, superiority, equivalence, noninferiority, exploratory)**

Methods: Participants, interventions, and outcomes

**Study setting Page 4, Line 39**	**9**	**Description of study settings (eg, community clinic, academic hospital) and list of countries where data will be collected. Reference to where list of study sites can be obtained**
**Eligibility criteria Page 4, Line 42**	**10**	**Inclusion and exclusion criteria for participants. If applicable, eligibility criteria for study centres and individuals who will perform the interventions (eg, surgeons, psychotherapists)**
**Interventions Page 5, Line 36**	**11a**	**Interventions for each group with sufficient detail to allow replication, including how and when they will be administered**
	**11b**	**Criteria for discontinuing or modifying allocated interventions for a given trial participant (eg, drug dose change in response to harms, participant request, or improving/worsening disease)**
	**11c**	**Strategies to improve adherence to intervention protocols, and any procedures for monitoring adherence (eg, drug tablet return, laboratory tests)**
	**11d**	**Relevant concomitant care and interventions that are permitted or prohibited during the trial**
**Outcomes Page 6, Line 8**	**12**	**Primary, secondary, and other outcomes, including the specific measurement variable (eg, systolic blood pressure), analysis metric (eg, change from baseline, final value, time to event), method of aggregation (eg, median, proportion), and time point for each outcome. Explanation of the clinical relevance of chosen efficacy and harm outcomes is strongly recommended**
**Participant timeline Page 5, Line 36. Figure 1**	**13**	**Time schedule of enrolment, interventions (including any run-ins and washouts), assessments, and visits for participants. A schematic diagram is highly recommended (see Figure)**
**Sample size Page 7, Line 11**	**14**	**Estimated number of participants needed to achieve study objectives and how it was determined, including clinical and statistical assumptions supporting any sample size calculations**
**Recruitment Page 7, Line 33**	**15**	**Strategies for achieving adequate participant enrolment to reach target sample size**

Methods: Assignment of interventions (for controlled trials)

Allocation:

For peer review only - http://bmjopen.bmj.com/site/about/guidelines.xhtml

**Sequence generation Figure 1**	**16a**	**Method of generating the allocation sequence (eg, computer-generated random numbers), and list of any factors for stratification. To reduce predictability of a random sequence, details of any planned restriction (eg, blocking) should be provided in a separate document that is unavailable to those who enrol participants or assign interventions**
**Allocation concealment mechanism Page 7, Line 48**	**16b**	**Mechanism of implementing the allocation sequence (eg, central telephone; sequentially numbered, opaque, sealed envelopes), describing any steps to conceal the sequence until interventions are assigned**
~~Implementation~~	~~16c~~	~~Who will generate the allocation sequence, who will enrol participants, and who will assign participants to interventions~~
**Blinding (masking) Page 8, Line 3**	**17a**	**Who will be blinded after assignment to interventions (eg, trial participants, care providers, outcome assessors, data analysts), and how**
	**17b**	**If blinded, circumstances under which unblinding is permissible, and procedure for revealing a participant's allocated intervention during the trial**
**Methods: Data collection, management, and analysis**		
**Data collection methods Page 5, Line 16 Page 5, Line 35**	**18a**	**Plans for assessment and collection of outcome, baseline, and other trial data, including any related processes to promote data quality (eg, duplicate measurements, training of assessors) and a description of study instruments (eg, questionnaires, laboratory tests) along with their reliability and validity, if known. Reference to where data collection forms can be found, if not in the protocol**
	~~18b~~	~~Plans to promote participant retention and complete follow-up, including list of any outcome data to be collected for participants who discontinue or deviate from intervention protocols~~
Data management **Page 8, Line 12**	19	Plans for data entry, coding, security, and storage, including any related processes to promote data quality (eg, double data entry; range checks for data values). Reference to where details of data management procedures can be found, if not in the protocol
**Statistical methods Page 8, Line 17**	**20a**	**Statistical methods for analysing primary and secondary outcomes. Reference to where other details of the statistical analysis plan can be found, if not in the protocol**
	~~20b~~	~~Methods for any additional analyses (eg, subgroup and adjusted analyses)~~
	~~20c~~	~~Definition of analysis population relating to protocol non-adherence (eg, as randomised analysis), and any statistical methods to handle missing data (eg, multiple imputation)~~

For peer review only - http://bmjopen.bmj.com/site/about/guidelines.xhtml

Methods: Monitoring

**Data monitoring Page 8, Line 44**	**21a**	**Composition of data monitoring committee (DMC); summary of its role and reporting structure; statement of whether it is independent from the sponsor and competing interests; and reference to where further details about its charter can be found, if not in the protocol. Alternatively, an explanation of why a DMC is not needed**
	**21b**	**Description of any interim analyses and stopping guidelines, including who will have access to these interim results and make the final decision to terminate the trial**
**Harms Page 8, Line 30**	**22**	**Plans for collecting, assessing, reporting, and managing solicited and spontaneously reported adverse events and other unintended effects of trial interventions or trial conduct**
**Auditing Page 8, Line 44**	**23**	**Frequency and procedures for auditing trial conduct, if any, and whether the process will be independent from investigators and the sponsor**

Ethics and dissemination

**Research ethics approval Page 9, Line 10**	**24**	**Plans for seeking research ethics committee/institutional review board (REC/IRB) approval**
**Protocol amendments Page 9 , Line 16**	**25**	**Plans for communicating important protocol modifications (eg, changes to eligibility criteria, outcomes, analyses) to relevant parties (eg, investigators, REC/IRBs, trial participants, trial registries, journals, regulators)**
**Consent or assent Page 9, Line 32**	**26a**	**Who will obtain informed consent or assent from potential trial participants or authorised surrogates, and how (see Item 32)**
	~~26b~~	~~Additional consent provisions for collection and use of participant data and biological specimens in ancillary studies, if applicable~~
**Confidentiality Page 9, Line 38**	**27**	**How personal information about potential and enrolled participants will be collected, shared, and maintained in order to protect confidentiality before, during, and after the trial**
**Declaration of interests Page 9, Line 46**	**28**	**Financial and other competing interests for principal investigators for the overall trial and each study site**
**Access to data Page 9, Line 50**	**29**	**Statement of who will have access to the final trial dataset, and disclosure of contractual agreements that limit such access for investigators**

For peer review only

**Ancillary and post-trial care Page 9, Line 25**	**30**	**Provisions, if any, for ancillary and post-trial care, and for compensation to those who suffer harm from trial participation**
**Dissemination policy Page 9, Line 53**	**31a**	**Plans for investigators and sponsor to communicate trial results to participants, healthcare professionals, the public, and other relevant groups (eg, via publication, reporting in results databases, or other data sharing arrangements), including any publication restrictions**
	~~31b~~	~~Authorship eligibility guidelines and any intended use of professional writers~~
	~~31c~~	~~Plans, if any, for granting public access to the full protocol, participant-level dataset, and statistical code~~
**Appendices**		
~~Informed consent materials~~	~~32~~	~~Model consent form and other related documentation given to participants and authorised surrogates~~
~~Biological specimens~~	~~33~~	~~Plans for collection, laboratory evaluation, and storage of biological specimens for genetic or molecular analysis in the current trial and for future use in ancillary studies, if applicable~~

*It is strongly recommended that this checklist be read in conjunction with the SPIRIT 2013 Explanation & Elaboration for important clarification on the items. Amendments to the protocol should be tracked and dated. The SPIRIT checklist is copyrighted by the SPIRIT Group under the Creative Commons "Attribution-NonCommercial-NoDerivs 3.0 Unported" license.

For peer review only
